# Variation of floristic diversity, community composition, endemism, and conservation status of tree species in tropical rainforests of Sri Lanka across a wide altitudinal gradient

**DOI:** 10.1038/s41598-024-52594-3

**Published:** 2024-01-24

**Authors:** Nimalka Sanjeewani, Dilum Samarasinghe, Himesh Jayasinghe, Kanishka Ukuwela, Asanga Wijetunga, Sampath Wahala, Janendra De Costa

**Affiliations:** 1 Postgraduate Institute of Agriculture, University of Peradeniya, Peradeniya, Sri Lanka; 2 Postgraduate Institute of Archaeology, University of Kelaniya, Colombo, Sri Lanka; 3 National Institute of Fundamental Studies, Kandy, Sri Lanka; 4 Department of Biological Science, Faculty of Applied Sciences, Rajarata University of Sri Lanka, Mihintale, Sri Lanka; 5 Department of Tourism Management, Faculty of Management Studies, Sabaragamuwa University of Sri Lanka, Belihul Oya, Sri Lanka; 6 Department of Crop Science, Faculty of Agriculture, University of Peradeniya, Peradeniya, Sri Lanka

**Keywords:** Ecology, Plant sciences, Climate sciences, Ecology

## Abstract

Tropical rainforests in Sri Lanka are biodiversity hotspots, which are sensitive to anthropogenic disturbance and long-term climate change. We assessed the diversity, endemism and conservation status of these rainforests across a wide altitudinal range (100–2200 m above sea level) via a complete census of all trees having ≥ 10 cm diameter at breast height in ten one-hectare permanent sampling plots. The numbers of tree families, genera and species and community-scale tree diversity decreased with increasing altitude. Tree diversity, species richness and total basal area per ha across the altitudinal range were positively associated with long-term means of maximum temperature, annual rainfall and solar irradiance. Percentage of endangered species increased with increasing altitude and was positively associated with cumulative maximum soil water deficit, day-night temperature difference and high anthropogenic disturbance. Percentage of endemic species was greater in the lowland rainforests than in high-altitude montane forests. Nearly 85% of the species were recorded in three or less plots, which indicated substantial altitudinal differentiation in their distributions. Less than 10 individuals were recorded in 41% of the endemic species and 45% of the native species, which underlined the need for urgent conservation efforts across the whole altitudinal range.

## Introduction

Tropical forests are the one of the most diverse terrestrial ecosystems on Earth^1^. Sustenance of biodiversity of tropical rainforests is threatened by several natural and anthropogenic processes^2^. Atmospheric warming, increased frequency of droughts and forest fires caused by climate change have changed the habitat environment of many plant species so that it no longer corresponds to their ecological niche^3,4^. Human activities such as deforestation, habitat fragmentation and over-exploitation have caused habitat loss for many flora and fauna in forest ecosystems^5^. Accordingly, the need for assessment of conservation needs at the ecosystem and taxon level is important and urgent^6^.

Altitudinal gradients provide an excellent natural setting for investigating ecosystem responses to long-term environmental change, which would otherwise necessitate long-term observation^7,8^. As air temperature decreases systematically with increasing altitude, a series of tropical forest plots along an altitudinal gradient represents forest communities which have evolved at different temperature regimes. Therefore, trends in community structure, diversity and endemism in forest plots along altitudinal gradients can provide valuable insights into how vegetation dynamics are influenced by long-term climate change, in which increasing air temperatures is a key feature^9^. In the tropical zone, the narrow temperature gradients that occur across latitudes do not lend themselves to detection of the influence of temperature variation on ecosystem processes in tropical rainforests^7^. In contrast, altitudinal gradients in the tropics offer sufficiently wide temperature ranges over shorter distances with the absence of temperature seasonality being an added advantage. Even though edaphic and some climatic variations (e.g. period of cloud immersion, incident UV radiation etc.) could still be present across tropical altitudinal gradients, careful selection of sampling sites in rainforests could allow detection of the influence of temperature change on ecosystem structure and dynamics^10^. Notably, the spatial variation of temperature that occurs across altitudinal gradients in tropical rainforests is of the same order of magnitude of the long-term temporal variation of temperature that they have experienced as part of climate change^11^. Therefore, the use of altitudinal gradients to gain insights into the possible responses of the dynamics of community structure and functioning to long-term climate change is an application of the ergodic principle with the assumption that the observed variation across space represents accumulated responses over time^12^.

The composition and diversity of an ecosystem at a given altitude represents the functional balance of long-term evolutionary processes that determine recruitment, growth, reproduction and mortality of different taxa and competition among them as shaped by the climate and soil at that altitude^13–15^. Furthermore, phylogenetic relationships among species have been shown to be a significant factor in determining community composition in the ecological niche as defined by a given altitude range. For example, Webb^16^ showed for a series of tropical rainforest plots in Borneo that taxa with greater phylogenetic relatedness are more likely to inhabit ecologically similar environments. On the other hand, Kembell and Hubbell^17^ observed both phylogenetic clustering and dispersion in tree species at spatial scales ranging from 10^2^ to 10^4^ m^2^ in a Neotropical rainforest in the Borro Colorado Island, Panama. Therefore, the composition and diversity of rainforest communities at different altitudes could be determined by the relative magnitudes of the forces of environmental filtering, niche differentiation and competitive exclusion^18,19^, of speciation, migration/dispersal and extinction rates^20^ (Hubbell, 2001) and their historical patterns^21^. Accordingly, it is highly likely that multiple processes operating across different spatial and temporal scales^22–24^ drive the community ecology of tropical rainforests across altitudinal gradients. Ecological succession could add a further layer of control on community composition and diversity because of differential survival and replacement of taxa. For example, Letcher et al.^25^ showed that phylogenetic relatedness of tree communities of Neotropical rainforests undergoing succession decreased with successional stage.

Anthropogenic climate change acts as a strong driver of change in the geographical range of tree species, community assembly and diversity of tropical rainforests by driving adaptive and evolutionary responses^26^. Climate change could cause shifts in the fundamental niche of a species, thus resulting in a shift in the geographical range of the species^27,28^. Furthermore, climate change could initiate adaptive responses in species to changes occurring in their habitat while also modifying interspecific biotic interactions such as competition, mutualism and trophic relationships^29–31^. Accordingly, the altitudinal range across which a given species is present shows its favourable ecological range, which is also an indication of a taxon’s resilience or vulnerability to environmental change, both gradual (e.g. climate change) and abrupt (e.g. deforestation, fire)^32^. As temperatures increase over time, migration of lowland-adapted taxa to higher altitudes and extinction of highland-adapted taxa can be expected in the absence of adaptive and evolutionary responses^32–38^.

The conservation status of a given species is a collective assessment based on its current population, ecological range and its probability of survival^39–42^. Accordingly, the natural and anthropogenic changes in the environment and habitat which are superimposed on the environmental gradients along altitudes have a substantial influence on the conservation status of a given species within an ecosystem. Tropical rainforests are a primary focus in conservation initiatives as they represent an ecosystem of high biodiversity threatened by natural and anthropogenic drivers. Therefore, in the present work, we focused on assessing the conservation status of woody plant taxa in the tropical rainforests of Sri Lanka, which in combination with forests in the Western Ghats of India, had been included in the 18 originally-designated biodiversity hotspots^1^ (Myers et al. 2000). The rainforests of Sri Lanka span an altitudinal range from 100 to 2200 m above mean sea level (asl) and include lowland wet-evergreen, lower-montane and upper-montane forests^43^. During the last three centuries, the rainforests in the South Asian Region have become increasingly threatened with deforestation and fragmentation^43,44^. Therefore, an assessment of the conservation status of their taxa along an altitudinal gradient provides a foundation for conservation efforts in a future changing climate.

Globally, there have been several studies of the variation of tropical rainforests along altitudinal gradients^8,36,45–51^. However, there is a paucity of such studies on the tropical forests of South Asia. Tropical rainforests of Sri Lanka and those of the Western Ghats share a common tectonic plate, known as the Deccan Plate, of southern Gondwana origin and their floristics is considered to be distinct from that of rainforests in the rest of tropical Asia^52^. Plate tectonics and continental drift have probably played a significant role in the floristics of the Sri Lankan tropical rainforests, especially of the lowland rainforests in the South–West. The Deccan Plate had been isolated for nearly 35 million years following the breakup of the Gondwana continent in the early Cretaceous times before colliding with the southern Laurasian continent in mid-Tertiary times^53^. This has allowed substantial mixing of the Deccan Gondwana flora with those from Southern Laurasia, Australia, New Guinea and South Pacific Islands^54–56^. In the recent phylogenetic classification of the world’s tropical forests^57^, Sri Lankan tropical rainforests have been classified into the Indo-Pacific floristic region.

Despite the absence of endemic families and the presence of only a few endemic genera, the rainforests in the lowlands of the South–West of Sri Lanka and on the western slope of the Central Highlands contain more than 700 endemic plant species^52^. At the lower to lower-mid altitudes up to 900 m above sea level, the characteristic tree species occupying the canopy stratum (35–40 m high) are *Dipterocarpus zeylanicus*, *D. hispidus, Mesua ferrea* and *Shorea trapezifolia*^43^. On the other hand, the sub-canopy is commonly occupied by *Cullenia rosayroana, C. zeylanica* and *Myristica dactyloides,* whereas *Xylopia championii* and *Garcinia hermonii* are the common understorey tree species in lowland rainforests of Sri Lanka. In the mid-altitude (900–1500 m) lower montane forests, the canopy stratum (20–25 m) is dominated by *Shorea gardneri*, *Calophyllum* spp., *Cryptocarya wightiana*, *Myristica dactyloides* and *Syzygium* spp. In the high-altitude (> 1500 m) montane forests with canopies of *ca.* 10 m tall, the dominant tree species are *Calophyllum walkeri*, *C. trapezifolium*, *Syzygium revolutum*, *S. rotundifolium*, *S. umbrosum*, *Symplocos cochinsinensis*, *Neolitsea fuscata*, *Cinnamomum ovalifolium*, *Litsea ovalifolia* and *Actinodaphne speciosa*^43,58,59^.

Our primary objective in this work was to assess the species diversity, vulnerability and conservation importance of the diverse tree communities of these tropical rainforests of Sri Lanka across their whole altitudinal range. We then aimed to determine the influence of climatic variation across the altitudinal range on tree diversity, endemism and conservation status. We carried out the above assessment by determining the (a) community-scale tree diversity; and (b) floristic composition of the tree communities in terms of endemism, conservation status, dominance and relative importance at family, genus and species levels along an altitudinal gradient. Because of the wide temperature gradient that occurs along tropical altitudinal gradients, the altitudinal variations in the above aspects of tree communities could provide indications of the vulnerability of tropical rainforests of Sri Lanka and South Asia to climate change. In accordance with the above, we sought answers to the following research questions on the altitudinal variation of tree diversity and forest structure in tropical rainforests in Sri Lanka: (1) Does the community composition, at the family, genus and species levels, and tree diversity show identifiable trends with altitude?; (2) Does the variation of climate with altitude influence the tree community composition and diversity?; (3) Does endemism at the tree species level in the rainforest communities vary with altitude and if so, what influence does climatic variation across altitudes have on endemism?; (4) Is there a discernible trend in the conservation status of tree species with altitude and if so, what have been the comparative influences of anthropogenic disturbance and climate on it?

## Results

### Variation of climate with altitude

The long-term annual total rainfall (R_F_) showed an overall decreasing trend with altitude,but plateaued around a minimum of 1900–2000 mm y^−1^ from 1800 m above sea level (asl) upwards (Table 1). The vapour pressure deficit (VPD) did not show an altitudinal trend. Long-term (1990–2018) means of monthly maximum soil water deficit (SWD_max_) and cumulative soil water deficit (CSWD_max_) increased with increasing altitude. Forests in permanent sampling plots (PSPs) located up to 1100 m asl did not experience any ‘dry’ months whereas those above 1100 m (i.e. RLG, HKG, PTG and HNP) experienced 1–3 ‘dry’ months. February is a ‘dry’ month in all four PSPs whereas March is a ‘dry’ month in three PSPs with RLG being the exception. The other ‘dry’ month was June (in HKG and PTG).

**Table 1:** Long-term (1970–2018) climate of the permanent sampling plots (PSPs).

PSP	Alt. (m)	T_avg_ (^O^C)	T_max_ (^O^C)	T_min_ (^O^C)	DTR (^O^C)	R_F_ (mm y^−1^)	S_R_ (MJ m^−2^ d^−1^)	V_P_ (kPa)	VPD (kPa)	W (ms^−1^)	SWD_max_ (mm month^−1^)	CSWD_max_ (mm)	No. of ‘dry’ months^†^
KDN1	117	26.40	29.52	23.57	5.95	3670	18.945	2.83	0.71	2.3	7.01	10.73	0
KDN2	174	26.18	29.14	22.98	6.16	3858	18.833	2.75	0.67	2.3	5.85	9.49	0
PTD2	509	24.15	27.49	20.99	6.50	3899	18.496	2.17	0.97	1.9	6.06	9.70	0
PTD1	618	23.82	26.99	20.65	6.34	3911	18.330	2.00	0.98	1.9	6.06	9.70	0
ENS1	1042	21.56	24.84	18.32	6.51	3130	17.910	2.00	0.58	2.2	17.58	23.71	0
ENS2	1065	21.56	24.84	18.32	6.51	3130	17.910	2.00	0.58	2.2	17.58	23.71	0
RLG	1668	19.65	23.40	15.82	7.58	2796	17.731	1.75	0.43	1.8	15.04	27.43	1 (F)
HKG	1804	17.05	20.67	13.48	7.19	2024	17.688	1.75	0.25	2.0	28.29	48.61	3 (F,M,Jn)
PTG	2080	15.56	19.14	11.98	7.15	1949	17.460	1.17	0.64	1.9	36.28	66.55	3 (F,M,Jn)
HNP	2132	14.75	18.32	11.34	6.98	2049	17.398	1.17	0.55	2.1	42.21	73.93	2 (F, M)

Alt., Altitude; Kanneliya Plot 1 (KDN1) and Plot 2 (KDN2), Sinharaja-Pitadeniya Plot 1 (PTD1) and Plot 2 (PTD2), Sinharaja-Enasalwatte Plot 1 (ENS1) and Plot 2 (ENS2), Rilagala (RLG), Hakgala (HKG), Piduruthalagala (PTG) and Horton Plains (HNP). T_mean_, Long-term annual average; T_max_, maximum and T_min_, minimum temperatures; DTR, Day-night temperature range; R_F_, Annual total rainfall; S_R_, Mean daily incident solar irradiance; Vp, Mean atmospheric vapour pressure; VPD, Vapour pressure deficit; W, Mean wind speed; SWD_max_, Maximum monthly soil water deficit; Cumulative SWD_max_, Maximum cumulative soil water deficit during consecutive months. ^†^A month is defined as a ‘dry’ month if the monthly rainfall is less than monthly evapotranspiration in more than 50% of the calculating period (1990–2018) and if its mean monthly soil water deficit over the calculating period exceeded 20 mm month^−1^. F-February, M-March, Jn-June.

### Forest structure and vegetation diversity

**Figure 1:**
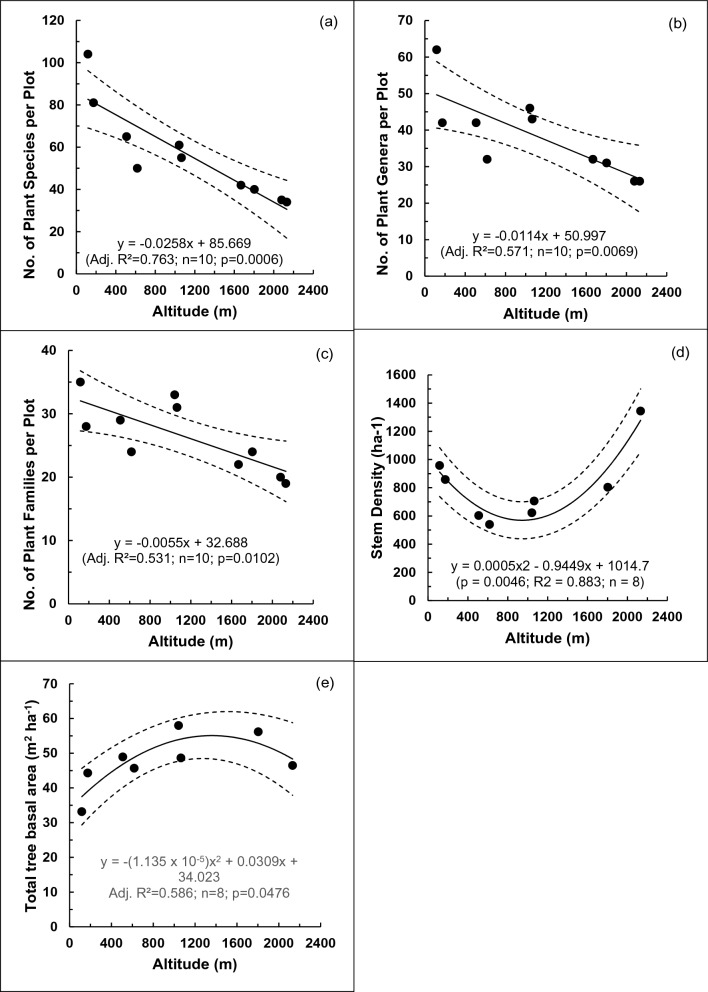
Variation of the numbers of: (**a**) plant species; (**b**) plant genera; (**c**) plant families and (**d**) stem density; (**e**) total tree basal area with altitude in permanent sampling plots in selected tropical rainforests in Sri Lanka. Dotted curves show the 95% confidence limits of the fitted linear (**a**–**c**) and polynomial (**d**, **e**) regressions. In (**d**) and (**e**) the curves have been fitted after excluding the two outliers at 1668 and 2080 m.

**Figure 2:**
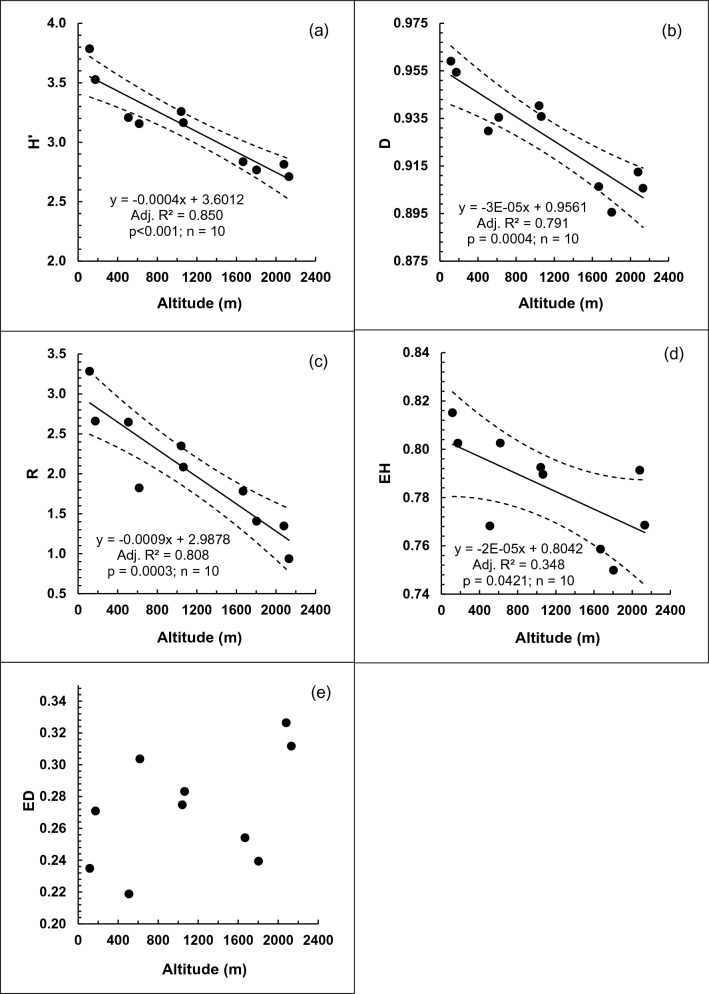
Variation of the (**a**) Shannon Weiner diversity index (H’), (**b**) Simpson’s diversity index (D), (**c**) Species richness R), (**d**) Shannon evenness (EH) and (**e**) Simpson’s evenness (ED) among the tropical rainforests in Sri Lanka along an altitudinal gradient.

In a total plot area of 10.851 ha, we enumerated 8027 trees having DBH ≥ 10 cm, belonging to 276 tree species from 145 genera and 69 families, with a total basal area of 445.37 m^2^. The numbers of species, genera and families decreased linearly (*p* < 0.05) with increasing altitude (Fig. 1a–c). Stem density decreased from low to mid-altitudes and then increased from mid- to high altitudes (Fig. 1d). This trend fitted a negative second-order polynomial function, which became significant (*p* < 0.05) when the two outlying data points at 1668 m and 2080 m were excluded. In contrast, total tree basal area showed a positive second-order polynomial trend with altitude with an estimated maximum of 55.08 m^2^ ha^−1^ at 1362 m (Fig. 1e). Shannon–Wiener (H’) and Simpson’s (D1) diversity indices and Menhinick’s species richness (R) decreased linearly (*p* < 0.001) with increasing altitude (Fig. 2a–c). Shannon–Wiener evenness of tree species also showed a linear decline (*p* < 0.05) with altitude. However, Simpson’s evenness did not show a significant trend (Fig. 2d–e).

### Floristic composition

Across the whole altitudinal range, the most dominant families were Dipterocarpaceae, Callophyllaceae, Malvaceae and Myrtaceae. However, Dipterocarpaceae and Malvaceae were observed only up to 1065 m whereas Callophyllaceae and Myrtaceae were present across the whole altitudinal range. Different altitude classes had different dominant plant families, viz*.* 0–400 m: Dipterocarpaceae, Malvaceae, Sapotaceae; 400–800 m: Dipterocarpaceae, Calophyllaceae, Malvaceae; 800–1200 m: Dipterocarpaceae, Clusiaceae, Myristicaceae; 1200–1800 m: Magnoliaceae, Sapindaceae, Lauraceae; Above 1800 m: Lauraceae, Symplocaceae, Myrtaceae.

**Figure 3:**
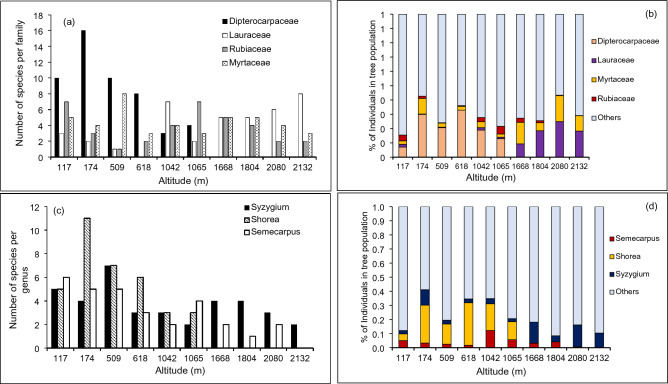
The four most diverse plant families (**a**) and the three most diverse genera (**c**) and their percentage contributions to the total tree population in each sampling plot (**b** and **d**) in selected tropical rainforests in Sri Lanka across an altitudinal gradient.

Myrtaceae, Dipterocarpaceae, Rubiaceae and Lauraceae were the most diverse families with 23, 22, 21 and 14 species respectively. At the lower and lower-mid altitudes, the diversity of Dipterocarpaceae was greater than those of the other three families. A similar superiority in diversity was shown by Lauraceae at altitudes above 2000 m. The diversity of Dipterocarpaceae was higher at the lower (i.e. < 400 m) and lower-mid (400–800 m) altitudes than at the mid altitudes (800–1200 m) (Fig. 3a). In contrast, Lauraceae showed a higher diversity at altitudes above 1600 m than at lower and lower-mid altitudes. Diversities of Rubiaceae and Myrtaceae did not show clear patterns of variation with altitude.

Myrtaceae was represented by 18 *Syzygium* species, four *Eugenia* species and one *Rhodomyrtus* species. Genus *Shorea* dominated the Dipterocarpaceae family with 14 species along with three from *Dipterocarpus*, two each from *Hopea* and *Stemonoporus* and one *Vateria* species. Family Lauraceae had five species from genus *Actinodaphne*, four from *Cinnamomum*, two each from *Neolitsea* and *Litsea* and one from *Cryptocarya*. Rubiaceae had a broader range of genera.

Across the whole altitudinal range, the most dominant genera were *Shorea*, *Syzygium* and *Allophylus*. Different genera were dominant at different altitude classes. *Shorea* was the most dominant genus in the altitude classes 0–400 m, 400–800 m and 800–1200 m. *Magnolia* was the most dominant genus at 1200–1800 m while *Symplocos* was the most dominant above 1800 m. The three most diverse genera were *Syzygium*, *Shorea* and *Semecarpus* with 18, 14 and 11 species respectively (Fig. 3c). *Shorea* was present only up to 1065 m whereas *Syzygium* and *Semecarpus* were present across the whole altitudinal range.

Despite their greater diversity in terms of the number of species recorded, contributions from the most diverse families and genera to the total tree number in the plots were relatively low (Fig. 3b and d). This meant that other less diverse plant families and genera contributed a greater proportion of individuals to the total tree population in the plots.

Across all plots, the most abundant plant family, in terms of the number of individuals, was Dipterocarpaceae. At different altitudes, different plant families were the most abundant. The families which contributed the highest percentage of individuals were Apocynaceae at 117 m, Dipterocarpaceae at 174 m, 618 m and 1042 m, Calophyllaceae at 509 m, Clusiaceae at 1065 m, Sapotaceae at 1668 m, Sapindaceae at 1804 m, Lauraceae at 2080 m and Symplocaceae at 2132.

**Figure 4:**
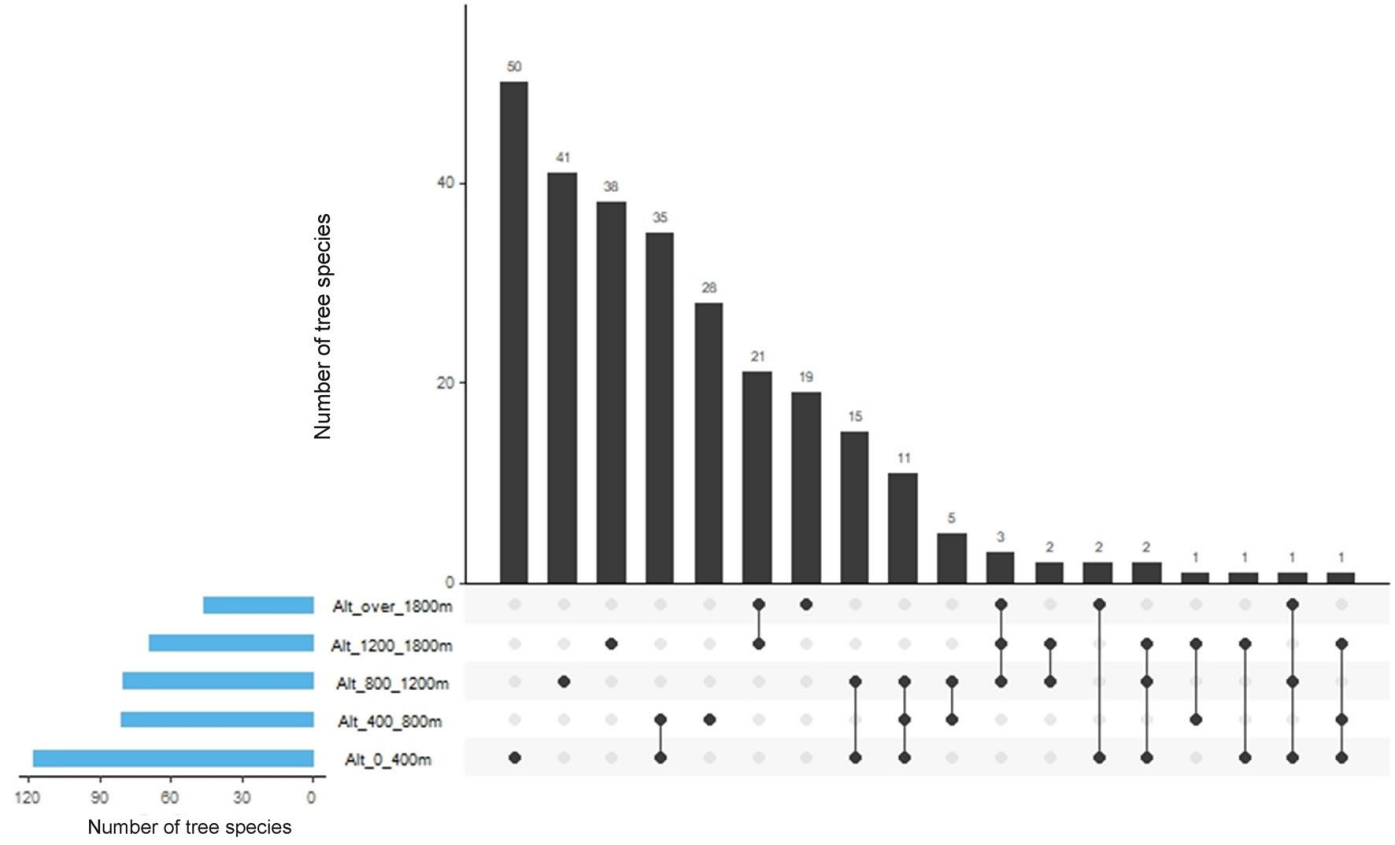
Differential distribution of tree species across the range of altitude classes. Vertical bars represent the number of species at a given altitude class or a range of altitude classes as shown along the horizontal axis. Horizontal bars on the bottom left represent the number of tree species recorded at each altitudinal class, which include the number of species with overlapping altitude ranges.

*Shorea* was the most abundant genus across all plots. *Shorea* was the most abundant genus at 174 m, 618 m and 1042 m (Fig. 4d). The most abundant genera were *Alstonia* at 117 m, *Mesua* at 509 m, *Garcinia* at 1065 m, *Palaquium* at 1668 m, *Allophylus* at 1804 m, *Neolitsea* at 2080 m and *Symplocos* at 2132 m.

The most dominant plant species differed in different altitude classes and belonged to different plant families (Table 2). Within the same altitude class also, the most abundant tree species differed in different sampling plots. Accordingly, at the individual plot level, the most abundant species were *Alstonia macrophylla* at 117 m, *Syzygium firmum* at 174 m, *Mesua thwaitesii* at 509 m, *Shorea affinis* at 618 m, *Shorea gardneri* at 1042 m, *Garcinia echinocarpa* at 1065 m, *Palaquium rubiginosum* at 1668 m, *Allophylus zeylanicus* at 1804 m, *Neolitsea fuscata* at 2080 m and *Symplocos bractealis* at 2132 m.

**Table 2:** The three most dominant species at different altitudinal classes along with their conservation status and endemism within one-hectare permanent sampling plots in tropical rainforests of Sri Lanka along an altitudinal gradient.

Altitude (m)	Species	Family	Conservation Status	Endemism	RD^†^	RF	RBA	IVI
0–400	*Cullenia rosayroana*	Malvaceae	LC	Endemic	7.47	1.08	10.72	19.27
	*Alstonia macrophylla*	Apocynaceae	NE	Exotic	10.25	0.54	6.92	17.72
	*Syzygium firmum*	Myrtaceae	LC	Native	4.56	1.08	9.24	14.89
400–800	*Shorea affinis*	Dipterocarpaceae	VU	Endemic	10.33	1.74	12.52	24.59
	*Durio ceylanicus*	Malvaceae	LC	Endemic	9.25	1.74	13.02	24.01
	*Mesua thwaitesii*	Calophyllaceae	LC	Endemic	11.95	1.74	4.99	18.68
800–1200	*Shorea gardneri*	Dipterocarpaceae	VU	Endemic	5.46	1.72	28.37	35.56
	*Shorea trapezifolia*	Dipterocarpaceae	VU	Endemic	7.10	1.72	16.00	24.82
	*Myristica dactyloides*	Myristicaceae	LC	Native	6.91	1.72	8.51	17.14
1200–1800^‡^	*Magnolia nilagirica*	Magnoliaceae	VU	Native	3.25	1.22	24.53	29.00
	*Allophylus zeylanicus*	Sapindaceae	LC	Endemic	17.22	1.22	6.25	24.68
	*Neolitsea fuscata*	Lauraceae	VU	Endemic	9.84	1.22	10.15	21.21
Above 1800	*Ilex walkeri*	Aquifoliaceae	LC	Native	10.98	2.90	14.56	28.44
	*Symplocos bractealis*	Symplocaceae	EN	Endemic	11.47	2.90	8.20	22.57
	*Rhododendron arboreum*	Ericaceae	VU	Endemic	10.98	1.45	8.91	21.34

^†^RD, Relative Density; RF, Relative Frequency; RBA, Relative Basal Area; IVI, Importance Value Index. EN, Endangered; VU, Vulnerable; LC, Least Concern; NE, Not evaluated. ^‡^The plot at HKG was included in this altitude category despite being at 1804 m.

There was only one species, *Actinodaphne albifrons*, which was present across the whole altitudinal range. There were only two species whose distribution traversed the whole altitudinal range (Fig. 4, Table S1). *Actinodaphne albifrons* was recorded in the lowest (0–400 m), highest (> 1800 m) and the mid- (800–1200 m) altitude ranges whereas *Elaeocarpus amoenus* was present in the lowest and highest altitude ranges. Both these species are endemics. A substantial percentage of the species were recorded in one or two altitudinal classes only (Tables S1 and S2). When considered in terms of the distribution across different permanent sampling plots also, a high percentage of the 276 tree species were present in three-or-lesser number of plots (Table 3). However, three species were recorded in six out of the 10 plots. These were *Cullenia rosayroana* and *Semecarpus gardneri* which were found in all plots from low (117 m) to mid (1065 m) altitudes and *Melicope lunu-ankenda* which was present from mid (1042 m) to high (2132 m) altitudes (Fig. S1). There were 154 tree species that are endemic to Sri Lanka while 100 species are native and four are exotic. Nearly one-third of the endemics, just over half of the natives and all exotics were found in only one plot (Table 3). Nearly 93% of endemics and 93% of the natives were found in one or two altitudinal classes (Table S3). Among the endemic and native species, 40.9% and 45% respectively recorded only 2–10 individual trees across all plots (Table S4). Notably, only one individual was found in 9.7% of the endemics and 21% of the natives. In contrast, more than 200 individuals were recorded from four endemic species (*Shorea affinis, Cullenia rosayroana, Symplocos bractealis,* and *Neolitsea fuscata*) and one native species (*Ilex walkeri*).

**Table 3:** Distribution and endemism of tree species found in one-hectare permanent sampling plots within tropical rainforests of Sri Lanka along an altitudinal gradient.

	Number of plots in which a species is found				
	one plot	2–3 plots	4–5 plots	6 plots	Total
Species total	117 (42.4%)^†^	117 (42.4%)	39 (14.1%)	3 (1.1%)	276
Endemic spp.	50 (32.5%)	73 (47.4%)	29 (18.8%)	2 (1.3%)	154 (55.8%)^‡^
Native spp.	51 (51.0%)	38 (38.0%)	10 (10.0%)	1 (1.0%)	100 (36.2%)
Exotic spp.	4 (100.0%)	0 (0.0%)	0 (0.0%)	0 (0.0%)	4 (1.4%)
Unidentified	12 (66.6%)	6 (33.3%)	0 (0.0%)	0 (0.0%)	18 (6.5%)

^†^% of total species in each category in terms of the number of plots present; ^‡^% of total species for each category in terms of endemism.

### Tree species endemic to Sri Lanka

The numbers of endemic species and individuals were greater than the corresponding numbers of native species and individuals in a majority of plots (Fig. S2). This difference was greater at lower altitudes. While the number of endemic species per plot showed a clear decline with increasing altitude, the number of native species per plot remained within a narrow range (10–30 species) across the whole range of altitudes. The number of endemic individuals per plot showed a negative second-order polynomial trend with altitude (Fig. S2a). The corresponding number of native individuals per plot showed an increasing trend with altitude (Fig. S2b).

**Figure 5:**
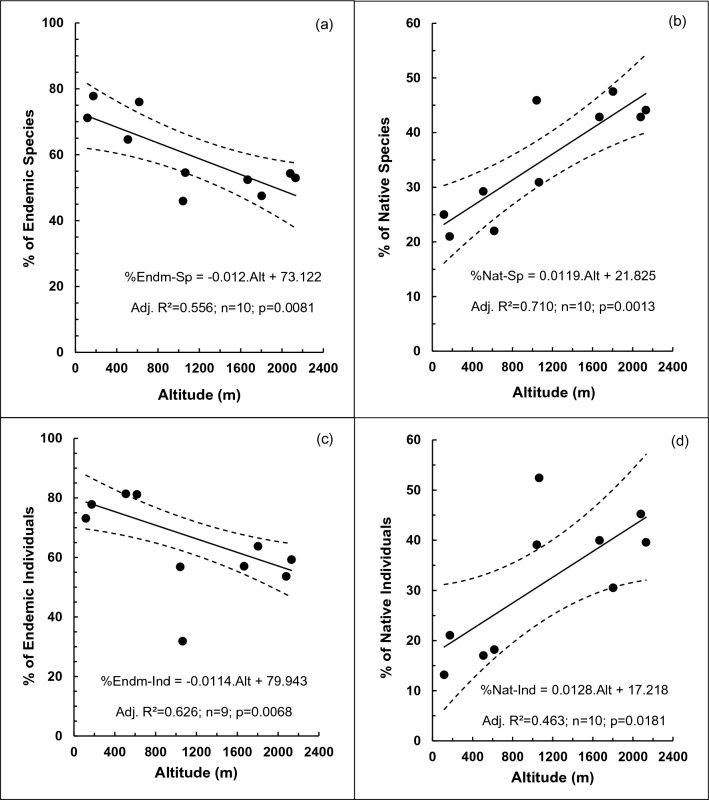
Variation of the per-plot percentages of: (**a**) endemic plant species; (**b**) native plant species; (**c**) endemic plant individuals and (**d**) native plant individuals with altitude in permanent sampling plots in selected tropical rainforests in Sri Lanka. Dotted curves show the 95% confidence limits of the fitted linear regressions. In (**c**) the linear regression has been done after excluding the outlier at 1065 m. Alt: Altitude.

The plot-wise percentage of endemic tree species decreased with increasing altitude (Fig. 5a). In contrast, the corresponding percentage of native tree species, which was almost 1-% endemics, increased with increasing altitude (Fig. 5b and d). At the individual tree level also, the percentage of endemics decreased (Fig. 5c) while the percentage of natives increased with increasing altitude.

### Conservation status of tree species

The conservation status, with reference to Sri Lanka as listed in the IUCN Red List, was found for 258 tree species. Accordingly, there was one species, *Eugenia fulva* (with four individuals), identified as ‘Critically Endangered Possibly Extinct, CR(PE)’ which was present in both plots at the mid-altitudes (1042 and 1065 m). There were three species which have been listed as ‘Critically Endangered (CR)’, *Palaquium zeylanicum* (32 individuals), *Syzygium kanneliyensis* (3 individuals) and *Eugenia sripadaense* (1 individual). These were found respectively at 117 and 174 m, 117 m and 1668 m. In fact, *Eugenia sripadaense* had hitherto been considered as a ‘point-endemic’ confined only to the Peak Wilderness Sanctuary in the Central Highlands of Sri Lanka (6°48′47″N, 80°29′04″E) (IUCN Red List of Threatened Species. Version 2022–2. https://www.iucnredlist.org. ISSN 2307–8235. https://www.iucnredlist.org/species/38011/10091773#assessment-information). This is the first record of its existence outside the above sanctuary. There were 40 species designated as ‘Endangered (EN)’, 79 listed as ‘Vulnerable (VU)’, 33 identified as ‘Near Threatened (NT)’ and 96 known as ‘Least Concern (LC)’. There were also six species which are categorized as ‘Not Evaluated (NE)’. Out of the three most dominant species identified for each altitude category (Table 2), at least two species were in the VU or EN conservation categories at altitudes above the 400–800 m category. With the exception of *Magnolia nilagirica* which is a native species, all other VU or EN species identified as dominant were endemics.

**Figure 6:**
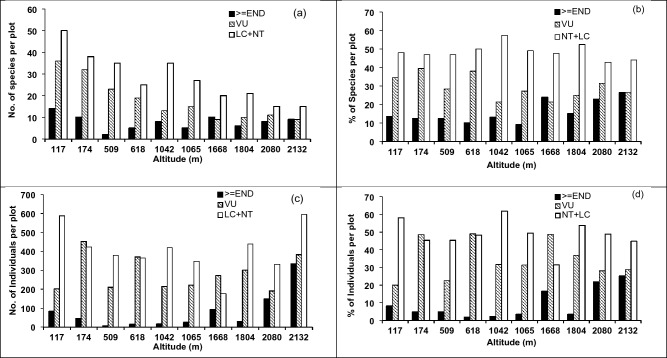
Conservation status of the (**a**) number of species (**b**) number of individuals (**c**) percentage of species (**d**) percentage of individuals in permanent sampling plots of selected tropical rainforests in Sri Lanka along an altitudinal gradient. >  = END: Endangered and above (i.e. Endangered, Critically-Endangered and Critically-Endangered and Nearly-Extinct); VU: Vulnerable; LC + NT: Least Concern and Near-Threatened.

At the plot level, the number endangered and above (≥ END), which included the conservation status categories CE-PE, CR and EN, did not show a clear variation pattern with altitude (Fig. 6a). However, the percentage of ≥ END tree species, out of the total number of species showed an increasing trend at upper altitudes (> 1100 m) (Fig. 6b). Within this increasing trend, the forest plot at 1668 m showed a substantial increase in the percentage of ≥ END species. In contrast to the number of ≥ END species, the number of tree species categorized as vulnerable (VU) decreased with increasing altitude. Both the absolute number of VU species and their percentage out of the total species in the plot were greater than the respective numbers and percentages of ≥ END species in 8 out of the 10 plots. The combined percentage of ≥ END and VU species, which represented the fraction of the tree population that required conservation effort, exceeded 33% in forest plots at all altitudes. It was greater than 45% in 7 out of the 10 plots while being greater than 50% in 3 out of those 7 plots. Two out of these 3 plots were located above 2000 m while the other was located at 174 m. The combined percentage of NT and LC, which represented the fraction of the tree population that does not require a conservation effort at present, ranged from 43% (at 2080 m) to 57% (1042 m).

Percentages of ≥ END and VU species showed significant negative quadratic trends with increasing altitude (Fig. S3). Notably, ≥ END species reached an estimated minimum at 654 m whereas the corresponding minimum for VU species occurred at the much higher altitude of 1531 m. The percentage of ≥ END individuals also showed a negative quadratic trend, with the estimated minimum occurring at 802 m. However, the corresponding percentage of VU individuals did not show a clear pattern. The percentage of NT + LC species showed a positive quadratic response to altitude, with the estimated maximum occurring at 1007 m. However, the corresponding percentage of NT + LC individuals did not show a clear trend.

### Influence of climate on floristic characteristics in the forest plots across the altitudinal gradient

**Figure 7:**
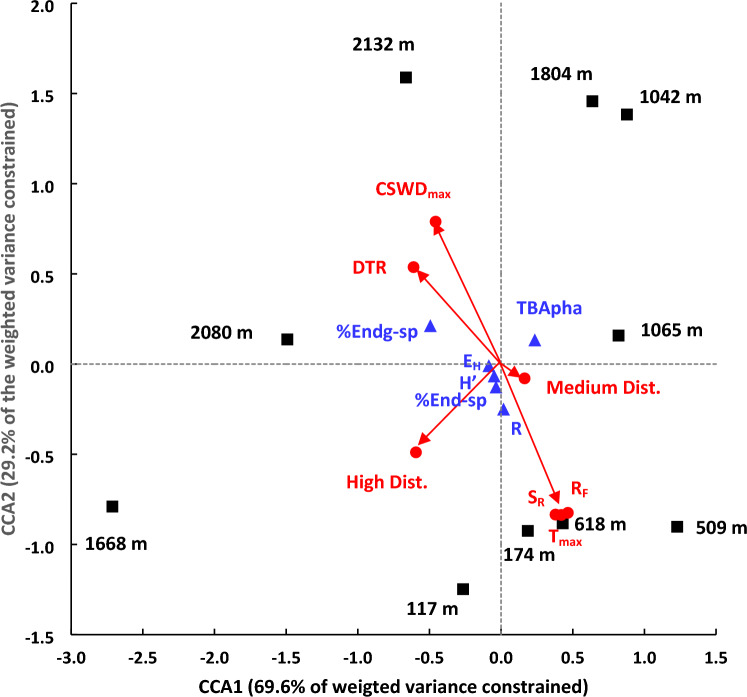
Tri-plot of the canonical correspondence analysis showing the influence of selected climatic variables (indicated in red) on selected floristic variables (in blue) as plotted along the two canonical correspondence axes (CCA) which constrained 98.8% of total inertia (i.e. weighted variance). Locations of the permanent sampling plots at different altitudes are shown in black. Floristic variables: H’ and E_H_—Shannon–Wiener diversity and evenness indices; R—Menhinick’s species richness; %End-sp—% of endemic species; %Endg-sp—% of endangered species. Climatic variables: T_max_—Maximum temperature; DTR—Day-night temperature range; R_F_—Rainfall; S_R_—Solar irradiance; CSWD_max_—Maximum cumulative soil water deficit. Disturbance status: High Dist.—High disturbance; Medium Dist.—Medium disturbance. Because of the close proximity of the data points of S_R_, T_max_ and R_F_, a common arrow has been drawn.

Canonical correspondence analysis (CCA) showed a significant (*p* < 0.05) relationship between the selected matrix of floristic variables and the combined matrix of climatic variables and disturbance status. The climatic variables, which were selected as contributing to the observed variance of floristic variables were long-term averages of maximum temperature (T_max_), day-night temperature range (DTR), rainfall (R_F_), maximum cumulative soil water deficit (CSWD_max_) and solar irradiance (S_R_). The climatic variables and the disturbance status explained 86% of the total inertia (i.e. weighted variance) (Table S5), thus indicating that climate and disturbance status exerted a significant influence on the forest floristic characteristics. Eigen vector analysis revealed that the first two axes explained 98.77% of the constrained inertia of the CCA (Table S6). All selected climatic variables contributed significantly to the observed variance of floristic characteristics. The inter-relationships among floristic characteristics of forests at different altitudes, climatic variables and disturbance status are shown in the CCA tri-plot (Fig. 7). For example, the percentage of endangered species (%Endg-sp) was positively associated with higher DTR and CSWD_max_, which were highest at the two highest altitude plots at 2080 m and 2132 m. On the other hand, TBApha was positively associated with T_max_, R_F_ and S_R_ and negatively associated with DTR and CSWD_max_. Similarly, the Menhinick’s species richness (R) and the percentage of endemic species (%End-sp) were positively associated with T_max_, R_F_ and S_R_ and negatively associated with DTR and CSWD_max_. On the other hand, the indices of species diversity (H’) and evenness (E_H_) were associated with lower DTR and CSWD_max_. A positive association was shown between high disturbance status and the percentage of endangered species. On the other hand, species diversity (H’) and richness (R) indices were positively associated with medium disturbance.

## Discussion

### Altitudinal trends of community composition and tree diversity

Our results show that community-scale tree diversity and species richness decrease with increasing altitude in the tropical rainforests in Sri Lanka, which are representative of those in South Asia. This trend agrees with similar trends observed by Gentry^45^, Aiba and Kitayama^47^, Homeier et al.^48^ and Cirimwami et al.^51^. In contrast, both Lieberman et al.^46^ and Clark et al.^8^ observed mid-altitude peaks of diversity while Girardin et al.^50^ also observed mid-altitude peaks numbers of tree families, genera and species. However, our results support the conclusion of Gentry^45^ that there is no ‘mid-elevation bulge’ in the diversity of tropical rainforests.

The observed decreases of tree diversity and species richness were caused by decreased numbers of families, genera and species with increasing altitude. These decreases indicate that environmental constraints at higher altitudes imposed a limitation on the colonization of tree species. For example, the reduction of mean annual air temperature from 26.4 to 14.75 °C across the altitudinal range (Table 1), probably exerted a restriction on the ecological range of most tree taxa of these tropical rainforests, which have evolved under and adapted to tropical climates. This agrees with Janzen’s^60^ hypothesis that thermophilic tropical species, which colonize the warmer lower altitudes, find it harder to colonize and survive at the cooler, higher altitudes. The substantially higher tree density at the highest altitude, which is typical of the high-altitude tropical montane forests in Sri Lanka, indicates that a smaller number of tree taxa are able to tolerate the environmental constraints (i.e. lower air temperatures, higher day-night temperature differential and higher soil water deficits) and proliferate at higher altitudes. A similar increase in stem density was observed at the highest altitude (2800 m) in the studies of Lieberman et al.^46^, Girardin et al.^50^ and Clark et al.^8^

Altitudinal ranges of the most diverse families contributed to the decreased community-scale diversity with altitude. The most diverse family in the lower altitudes (Dipterocarpaceae) was more diverse (i.e. a greater number of species) than the most diverse families in the higher altitudes (Lauraceae, Rubiaceae and Myrtaceae) (Fig. 3a), thus driving the reduction in diversity indices with altitude. Furthermore, whereas distribution of the latter three families extended into the lower altitudes, the distribution of Dipterocarpacea did not extend up to the higher altitudes. Similar variations in the most diverse plant genera (Fig. 3b) have further contributed to this trend. The most diverse genus at the lower altitudes, *Shorea*, had higher species numbers at low altitudes than the most diverse genera at the higher altitudes, *Syzygium* and *Semecarpus*. Furthermore, whereas *Syzygium* and *Semecarpus* extended to lower altitudes and increased the diversity there, *Shorea* did not extend beyond mid altitudes. Lieberman et al.^46^ and Aiba and Kitayama^47^ also observed changes in the distribution of families across altitudes. Agreeing with our observations, Aiba and Kitayama^47^ also observed Dipterocarpaceae and mostly *Shorea* species dominating their lower altitude (700 m) plots whereas Myrtaceae and *Syzygium* were among the dominant families and genera at higher altitudes respectively.

The observation that 42.4% of the 276 species recorded were present in only one plot while 84.8% were present in only three-or-lesser number of plots (Table 3) indicates a high degree of niche differentiation. However, the respective distributions of the three species which had the broadest altitudinal range (Fig. S1) show that a small minority of tree species were able to colonize a wider range of climates and soils.

### Variation of conservation status with altitude

**Figure 8:**
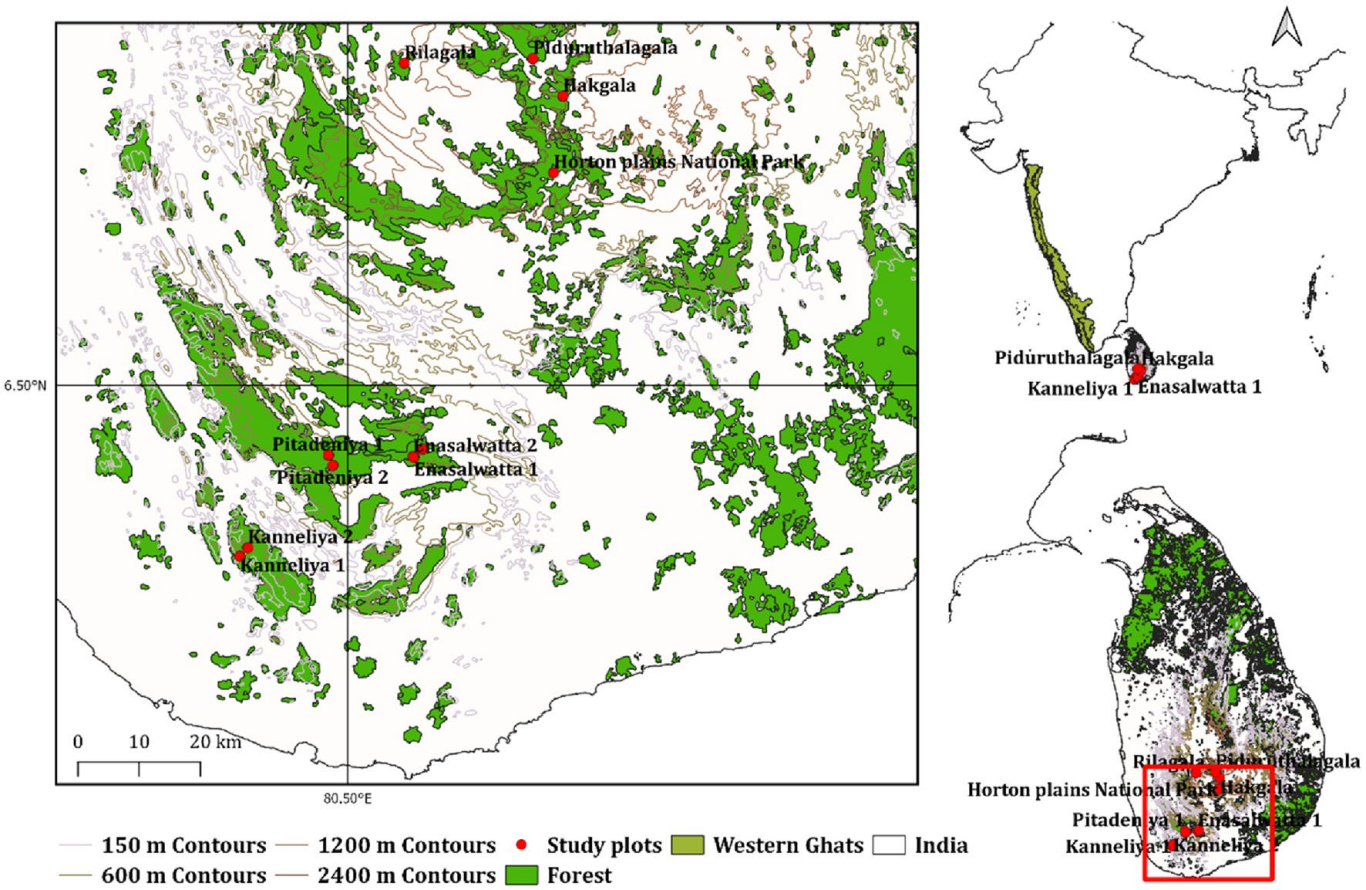
Permanent sampling plots of the present study located at different altitudes in tropical rain forests of Sri Lanka. Kanneliya Plot 1 (KDN1 at 117 m asl) and Plot 2 (KDN2—174 m), Sinharaja-Pitadeniya Plot 1 (PTD1—618 m) and Plot 2 (PTD2—509 m), Sinharaja-Enasalwatte Plot 1 (ENS1—1042 m) and Plot 2 (ENS 2—1065 m), Rilagala (RLG—1668 m), Hakgala (HKG—1804 m), Piduruthalagala (PTG—2080 m) and Horton Plains (HNP—2132 m).

Conservation status of tree species in our plots indicates the high vulnerability for extinction for a majority tree species in these tropical rainforests. Of particular concern is the increasing trend of ≥ END species at altitudes above 650 m (Fig. 6 and Fig. S3a). The tree species that colonize the mid- to upper altitude ranges, especially those on ridges and mountain tops, are confined to a narrow geographical range. This is one of the reasons for the higher percentage of ≥ END species at higher altitudes. The lowland rainforests in the South–West of Sri Lanka (Fig. 8) have undergone fragmentation^43^ due to human encroachment. On the other hand, the high-altitude montane forests in the Central Highlands have remained less affected by fragmentation due to multiple factors. These include physical topographical barriers, colder climate and greater distance from human settlements which are sparsely populated in comparison to lowland forests in the South–West. Results of the CCA demonstrate the influence of anthropogenic disturbance on %Endg-sp as well (Fig. 7). High anthropogenic disturbance often causes habitat fragmentation^61,62^, thus making seed dispersal and propagation of the populations of endangered species more difficult. This will increase the probability of extinction for many tree species of Sri Lankan rainforests, which have shown a high degree of niche differentiation. Our results on the association between anthropogenic disturbance and increased conservation need are in accordance with the findings of Barlow et al.^63^, Betts et al.^64^ and Feng et al.^65^.

In an analysis of long-term temperature in different climatic zones of Sri Lanka, De Costa^66^ showed that the central highlands, where the upper-mid and high-altitude forest plots of the present study are located, has been the region which has experienced the highest rate of increase in the annual mean air temperature and the clearest and fastest reduction of annual total precipitation. There is no direct evidence of this long-term increase in temperature and decrease of precipitation influencing the abundance and distribution of tree species in the montane forests of Sri Lanka. However, the possible role of climate change (e.g. increasing temperatures causing higher evapotranspiration rates, which when combined with decreasing precipitation, inducing greater soil water deficits and stronger water stress in trees) in the survival and conservation status of high-altitude tree species merits further investigation. This has been confirmed by the results of the CCA, which showed a positive association between %Endg-sp and CSWD_max_.

In this regard, it may be pertinent to note that parts of the upper montane forest in the highest-altitude location (i.e. Horton Plains) of the present study has shown symptoms of dieback since the mid-1970s^67^. Notably, based on analysis of rainfall records dating back from 1960s, Werner^67^ speculated that increased frequency of ‘dry’ months (i.e. < 30 mm month^−1^ rainfall) in the 1970s, especially in 1976, could have triggered the initial dieback.

### Variation of endemism with altitude

The high degree of endemism among the tree communities at the lower altitudes (Fig. S2) and its observed decrease with increasing altitude (Fig. 5a and b) indicate that most endemics have probably evolved under and adapted to warmer humid tropical climates. A reduction of the percentage of endemics with increasing altitude (Fig. 5b), in parallel with the reduction of total species number (Fig. 1a), demonstrates an inability of the lowland-adapted species to establish at higher altitudes or long-term extinction of endemic species with increasing altitude^68^. The observation that more than 50% of the endemic species have 10-or-less individuals across the whole range of plots and altitudes (Table S4) not only highlights their extreme niche differentiation, but also their vulnerability to extinction. Accordingly, urgent conservation efforts are needed for these rare endemics.

The positive association between the percentage of endemic species (%End-sp) and several climatic variables (i.e. R_F_, T_max_ and S_R_) shown in the CCA show that climatic variation has exerted an appreciable control on endemism in the tropical rainforests in Sri Lanka. This agrees with the observations of Irl et al.^69^ who concluded that both topography and climate combine to drive plant species diversity and endemism. While topographic heterogeneities could create insular microenvironments to induce greater speciation rates, higher rainfall and solar irradiance levels could ensure survival of the new species by stimulating their growth. On the other hand, as indicated by the negative association between the percentage of endemic species and CSWD_max_ increasing soil water deficits could decrease the survival of the new plant species that may arise in fragmented and isolated microenvironments in these rainforests. This is consistent with the conclusion of Hagen et al.^70^ who attributed increasing long-term aridity during the Cenozoic to the lower plant diversity in the Afrotropical rainforests as compared to that of Neotropical and Indomalayan rainforests.

In view of the greater magnitude of long-term climate change in the Central Highlands of Sri Lanka^66^, the observed reduction of endemic species at higher altitudes (Fig. 5 and S2) agrees with the conclusion of Jansson^71^ that there would be fewer endemic species in areas which have experienced greater historical climate change. Furthermore, the lower percentage of endemic species at higher altitudes in our study is consistent with the conclusion of Sandel et al.^72^ that endemism is lower in regions of higher climate change velocity. However, it is highly likely that greater endemism in the tree communities in the lowland rainforests of Sri Lanka has its roots in the deeper underlying processes and phenomena operating over longer time scales than anthropogenic climate change. For example, phylogenetic analyses of palms, a family confined largely to tropical rainforests (TRFs), have shown that evolutionary processes initiated during the mid-Cretaceous period have contributed to the current floristic diversity of TRFs^73^. According to Ashton and Gunatilleke^52^, the lowland rainforests in the South–West of Sri Lanka is the only forest region which has an aseasonal perhumid tropical climate in the tropical rainforest axis between Western Malaysia (i.e. Sumatra, Malaya, Borneo and the Philippines) and Eastern Madagascar. Accordingly, Ashton and Gunatilleke^52^ attributes the high endemism observed in the lowland rainforests of South–West Sri Lanka to habitat restriction caused by local heterogeneities due to the presence of ridge tops, mid-slopes and deep valleys and fragmentation caused by land use conversion to agriculture during the last two centuries. Such fragmentation probably accentuated the limitations imposed by physical barriers (e.g. ridge tops) and poor seed dispersal capabilities. Interestingly, the two floristic regions with the highest endemicity as identified by Ashton and Gunatilleke^52^ have the wettest climate which is least seasonal. This indicates that local heterogeneities and habitat fragmentation have had a stronger influence than climatic constraints on increased speciation and endemicity in the lowland rainforests in South–West Sri Lanka.

The floristic similarities between the upper montane forests of Sri Lanka and South–Western India, which are considered as one biogeographical unit^52,58^, probably contributed to the lower percentage of endemic species in the upper-altitude forests of the present study. The two forests are on the western and eastern edges of the Deccan Plate, on mountains arising from its collision with Southern Eurasia in the Cretaceous and Miocene^52,53,74^. Ashton and Gunatilleke^52^ argue that during periods of glaciation, greater land connectivity between these two upper montane forests would have facilitated mixing of these cold-adapted flora which had a greater probability of surviving glaciation. Ashton and Gunatilleke^52^ also argues that flora of Gondwana origin probably experienced cooler temperate climates because of the ancient supercontinent’s southern latitudinal location. When the Deccan Plate moved towards northern latitudes prior to and after its collision with the Eurasian Plate, the warmer tropical climates, especially at the lower altitudes, may have initiated adaptive and evolutionary processes ultimately leading to greater speciation and higher endemicity in the lowlands. This is consistent with our observation that the most diverse families and genera in the higher altitudes extended their distribution to the lower altitudes while the most diverse families and genera in the lower altitudes did not extend their distribution to the higher altitudes.

In contrast to the high-altitude montane forests, the lowland rainforests of Sri Lanka are not considered as an extension of the tropical evergreen forests of the South-West of India^55^ with the mixed Dipterocarp forests of the western Malaysia being their closest counterparts in the South Asian region. Several factors could have contributed to the increased endemism among the woody tree species in this region. It is notable that lowland rainforests are distributed across a lower area (*ca.* 141,000 ha) than the montane forests (*ca.* 312,000 ha)^43^. This reflects the greater fragmentation of lowland rainforests, which may have increased the possibility of creating insular environments, thus leading to greater endemism.

The pattern of decreasing endemism with increasing altitude observed in our study is contrary to the pattern of increasing endemism with increasing altitude at the global scale^75^ and in oceanic islands^76^ or the ‘hump-shaped’ pattern of greater endemism at mid- to higher altitudes along mountains^77,78^. However, in a study of taxon-specific altitudinal patterns of endemism, Kessler^79^ concluded that different patterns exist for different taxa depending on taxon-specific ecological traits such as life form, reproductive and dispersal mechanisms and competitive ability. When all taxa were pooled, Kessler^79^ observed the highest endemism in the narrowest and most fragmented altitudes. Furthermore, Barajas-Barbosa et al.^80^ concluded that environmental heterogeneity is a major driver of endemism of vascular plants in oceanic islands. The highest endemism observed in the present study in the lowland rainforests where fragmentation was higher, and the area occupied was lower agrees with the conclusions of Kessler^79^ and Barajas-Barbosa et al.^80^. This is consistent with the conclusion of Steinbauer et al.^75^ that per-species speciation rate and endemism increase with increasing geographical isolation. Following a field study of selected floristic regions of Sri Lanka, Gunatilleke and Ashton^55^ concluded that the endemics have a poor capacity to re-invade gaps in the forest created by fragmentation. This could have increased the probability of speciation and consequently the percentage of endemic species at the lower altitudes.

### Control of community composition, structure, and tree diversity in tropical rainforests of Sri Lanka by climate and anthropogenic disturbance

Canonical correspondence analysis showed that climate and anthropogenic disturbance combine to exert almost total control (i.e. first two axes explaining nearly 99% of the constrained variation in vegetation properties) on the constrained variation of community ecology of tropical rainforests of Sri Lanka across their altitudinal range of occurrence. Increasing soil water deficits (SWD) and day-night temperature ranges (DTR) and high levels of anthropogenic disturbance are shown to be major drivers of increased percentage of endangered tree species in tropical rainforests of Sri Lanka. There is strong evidence that all three of the above drivers are likely to be strengthened in the near- and medium-term future, both globally and locally^2,9,43,63,66,81^. Our analysis shows that the two climatic drivers increase with increasing altitude across the distribution range of the Sri Lankan rainforests. Therefore, while greater conservation efforts are needed in the future across the whole altitudinal range, montane forests in the higher altitudes, arguably, need to be accorded greater priority. However, an equally valid argument could be advocated for focussing a greater conservation effort on the lowland rainforests in view of their greater endemism, niche differentiation and habitat fragmentation.

In contrast to the negative impacts of CSWD_max_ and DTR, the CCA reveals positive impacts of T_max_, R_F_ and S_R_ on forest structure and diversity. These three climatic variables promote plant growth and thereby facilitates greater tree basal area per ha. By increasing the probability of seedling survival, higher T_max_, R_F_ and S_R_ could increase diversity of tree species, especially in the highly fragmented and specialized microenvironments. This is particularly relevant because the majority of the observed tree species have shown a high degree of zonation and niche differentiation. Furthermore, the positive impacts of increased T_max_, R_F_ and S_R_ on tree diversity and basal area could be greater in the higher altitudes where lower T_max_, R_F_ and S_R_ (Table 1) limit seedling survival and tree growth.

Whereas a high degree of anthropogenic disturbance is shown to have a negative impact on the structure and diversity of forest communities, CCA revealed a positive association between diversity and a medium level of disturbance. In our plots, removal of a limited number of saplings and signs of minor human activities constituted medium disturbance. As such, a medium level of disturbance could induce greater species richness and diversity by allowing greater radiation penetration to the forest floor and activating the soil seed bank. In such a situation, the taller trees in the community provide the shade necessary for the germinating seedlings to survive and grow.

## Methods

### Study sites and field work

We conducted this work in ten 1 ha (100 m × 100 m) permanent sampling plots (PSPs) in tropical lowland and montane rainforests in Southwestern and Central Sri Lanka. The PSPs were established along an altitudinal gradient from 117 to 2132 m above sea level (asl) (Fig. 8) in the Kanneliya Forest Reserve (KDN1 and KDN2), Pitadeniya (PTD1 and PTD2) and Enasalwatte (ENS1 and ENS2) in the Sinharaja Man and Biosphere Reserve, Rilagala Forest Reserve (RLG), Hakgala Strict Nature Reserve (HKG), Piduruthalagala Forest Reserve (PTG) and Horton Plains National Park (HNP). At present, all these forests are strictly protected by statute. However, the Kanneliya Forest Reserve had been subjected to selective logging from mid-1960s to late-1970s. Geographic details of the PSPs are given Table S7. Long-term average climate of the PSPs is given Table 1. A detailed analysis of the variation of key soil properties in the PSPs is given in De Costa et al.^82^.

We enumerated the PSPs from January 2019 to December 2019. All plants with woody stems with a diameter at breast height (DBH) (Height = 1.3 m) ≥ 10 cm were measured and tagged. The standardized census protocols (http://www.rainfor.org/en/manuals) used by Phillips et al.^83^ and Baker et al.^84^ were followed when measuring the DBH of trees. Taxonomic identification was done using standard keys and the reference collection at the National Herbarium of Sri Lanka at Royal Botanical Gardens, Peradeniya^85,86^. Conservation status and endemism of plant species were determined using the National Red List 2012^87,88^. Here, species conservation status is divided into nine categories as Extinct (EX), Extinct in The Wild (EW), Critically Endangered (CR), Endangered (EN), Vulnerable (VU), Near Threatened (NT), Least Concern (LC), Data Deficient (DD) and Not Evaluated (NE). In the National Red List in Sri Lanka, an additional tag, but not an extra category, as Critically Endangered ‘Possibly Extinct’ CR (PE) has been included^87^.

### Computation of vegetation indices and classification of conservation status

The overall dominance of tree species was quantified by the Importance Value Index (IVI)^89^ calculated as the sum of relative density (RDen), relative frequency (RFreq) and relative dominance (RDom) as,

1 $$RDen=\frac{Number\, of\, individuals\, of\, a\, tree\, species\, per\, unit\, PSP\, area}{Number\, of\, individuals\, of\, all\, tree\, species\, per\, unit\, PSP\, area }\times 100$$

2 $$RFreq=\frac{Number\, of\, PSPs\, in\, which\, a\, tree\, species\, is\, recorded}{Total\, number\, of\, PSPs}\times 100$$

3 $$RDom=\frac{Total\, basal\, area\, of\, a\, tree\, species\, in\, a\, PSP}{Total\, basal\, area\, of\, all\, tree\, species\, in\, the\, PSP}\times 100$$

Shannon–Wiener (H’) and Simpson’s (D) diversity and evenness indices and Menhinick’s index of species richness (R) (i.e. the number of species in a given plot divided by the square root of the total number of individuals in the plot) were calculated according to Shannon^90^, Simpson^91^ and Menhinick^92^ as,

4 $$H^{\prime}=-\sum [\left(\frac{ni}{N}\right)\times {log}_{e}\left(\frac{ni}{N}\right) ]$$

5 $$D=1-SI=1- \frac{\sum {n}_{i} \left({n}_{i}-1\right)}{N\left(N-1\right)}$$

6 $$D=1-\sum P{i}^{2}$$

7 $$R=\frac{S}{\surd N}$$

where n_i_ is the number of individuals of ith species, N the total number of individuals, Pi = Proportion of individuals of ith species, S = total number of species at the site.

Shannon–Wiener Evenness (E_H_) and Simpson’s Evenness (E_D_) indices were computed as,

8 $${E}_{H}=\frac{{H}^{\prime}}{{H}_{m}^{\prime}}$$

9 $${E}_{D}=\frac{1-SI}{1-{SI}_{m}}$$

where H’_m_ and SI_m_ are the maximum values of H’ and SI^93^.

Percentages of endemic, native and exotic species in each PSP were calculated separately based on the numbers of species and individuals. Species belonging to conservation categories CR (PE), CR and EN were pooled and designated as ‘endangered (≥ END)’, which along with species classified as VU, required the most urgent and highest effort in conservation. Species classified as NT and LC were also pooled as ‘NT + LC’ and considered as requiring the least conservation effort.

### Characterization of the climatic variation across the altitudinal gradient

Long-term (1970–2018) historical climatic data for the locations of the PSPs were obtained from the global climatic databases WorldClim 2^94^ and CRU-TS-4.03, bias corrected with WorldClim 2.1^95^. Monthly averages of mean (T_mean_), maximum (T_max_) and minimum (T_min_) air temperatures, daily solar irradiance (S_R_), total rainfall (R_F_), vapour pressure (V_P_), and wind speed (W) were obtained for the period from 1970 to 2018. The monthly day-night temperature range (DTR) was computed as the difference between monthly means of T_max_ and T_min_ of each month. Monthly mean vapour pressure deficit (VPD) was computed as the difference between monthly mean saturation vapour pressure (SVP) and monthly mean actual vapour pressure (VP) according to Allen et al.^96^. Here, saturation vapour pressure was computed using the relationship between SVP and temperature^97^. Monthly mean SVP was computed as the mean of SVPs computed based on T_max_ and T_mean_. Vapour pressure deficit is a measure of atmospheric dryness and exerts direct influences on stomatal conductance^98–100^, transpiration^101^, photosynthesis ^102^ and growth^103^ of forest communities.

Annual means of T_min_, T_max_, T_mean_, DTR, VPD, S_R_ and W during the above period were calculated using their respective monthly means in each year. The annual means were averaged to compute their long-term averages for the respective PSPs. Mean annual R_F_ for the 1970–2018 period was computed by cumulating the monthly totals of each year and averaging them over the 49-year period. Soil water deficit (SWD) was computed separately for each PSP on a monthly basis for the period from January 1990 to December 2018, using the soil water deficit model of Malhi and Wright^104^. For each PSP, the maximum monthly SWD (SWD_max_) and maximum cumulative SWD (CSWD_max_) were computed as indices of climatological drought. The model computes SWD on a monthly basis as the difference between monthly rainfall and evapotranspiration, while taking in to account their possible within-month variation. When SWD increased continuously during successive months, the respective monthly SWDs were cumulated to compute cumulative SWD (CSWD) until the cumulative SWD was brought back to zero by rainfall in the following month/s. For each month of the year, monthly means of SWD and CSWD were computed by averaging the respective monthly values of the 29-year period from 1990 to 2018. The maximum mean monthly values of SWD and CSWD for each PSP were taken as SWD_max_ and CSWD_max_. Frequency and magnitude of droughts experienced by forests in the PSPs were assessed by identifying the number of ‘dry’ months based on two criteria. The first criterion quantified the frequency of months experiencing a water deficit. A ‘dry’ month was defined as one in which monthly rainfall was lower than its evapotranspiration in more than 50% of years during the 1990–2018 period. The second criterion quantified the magnitude of water deficit. A month in which mean SWD exceeded a minimum threshold of 20 mm month^−1^ was defined as ‘dry’. To be classified as a ‘dry’ month, both criteria had to be satisfied.

### Classification of the anthropogenic disturbance in the forest plots

We classified the disturbance status of the forest plots as ‘high’, ‘medium’ and ‘low’ on the basis of the presence/absence of exotic tree species, past logging history (in KDN1 and RLG), proximity to human settlements, observed disturbance in the plots (e.g. cutting of small trees, removal litter traps etc.) during the study period and possible human interactions (e.g. walking paths through the forest for extraction of non-timber forest products etc.) (Table S8).

### Statistical analysis

Altitudinal trends of the measured and calculated variables were determined by linear and quadratic regression analysis using SAS Studio^105^. Canonical correspondence analysis (CCA) was used to determine the influence of climatic variables and anthropogenic disturbance on selected forest floristic variables. The floristic variables were purposively selected to represent different aspects of our rainforest plots (i.e. structure, diversity, evenness, endemism and conservation status). Accordingly, the selected floristic variables were total basal area per ha (TBApha), Menhinick’s species richness (R), Shannon–Wiener indices of species diversity (H’) and evenness (E_H_) and the percentages of endemic species (%End-Sp) and ‘endangered’ (≥ END) species (%Endg-Sp). Similarly, the climatic variables were also selected purposively to represent different aspects of the climatic variation across the altitudinal gradient of our plots (i.e. temperature regime, water availability and solar irradiance). As such, the selected climatic variables were T_max_, DTR, R_F_, CSWD_max_ and S_R_. The status of anthropogenic disturbance was incorporated in the CCA as three discrete qualitative variables, viz. ‘high’, ‘medium’ and ‘low’, as determined based on the criteria given in Table S8. Significance of the CCA model was assessed using permutation tests^106^. Forward selection procedure was used to identify significant environmental variables for inclusion in the CCA model.

### Supplementary Information

Supplementary Information.

## Data Availability

The datasets generated during the current study are available from the corresponding author and Nimalka Sanjeewani on reasonable request.
