# Supplementary material for: Variation of floristic diversity, community composition, endemism, and conservation status of tree species in tropical rainforests of Sri Lanka across a wide altitudinal gradient
Source: Sci Rep. 2024 Jan 24;14:2090. doi: 10.1038/s41598-024-52594-3 (PMC10808289; doi:10.1038/s41598-024-52594-3)
Supplement: Supplementary file 1 — Supplementary Information. [file 41598_2024_52594_MOESM1_ESM.pdf]

Journal

Scientific Reports

Title

Variation of floristic diversity, community composition, endemism, and conservation status of tree species in tropical rainforests of Sri Lanka across a wide altitudinal gradient

Authors

Nimalka Sanjeewani[1,7], Dilum Samarasinghe[2], Himesh Jayasinghe[3], Kanishka Ukuwela[4], Asanga Wijetunga[4], Sampath Wahala[5], and Janendra De Costa[6] http://orcid.org/0000-0003-1923-0992 (Corresponding Author)

Affiliations

[1]Postgraduate Institute of Agriculture, University of Peradeniya, Sri Lanka

[2]Postgraduate Institute of Archaeology, University of Kelaniya, Sri Lanka

[3]National Institute of Fundamental Studies, Kandy, Sri Lanka

[4]Department of Biological Science, Faculty of Applied Sciences, Rajarata University of Sri Lanka, Sri Lanka

[5]Department of Tourism Management, Faculty of Management Studies, Sabaragamuwa University of Sri Lanka

[6]Department of Crop Science, Faculty of Agriculture, University of Peradeniya, Sri Lanka

Table S1. Complete list of tree species along with their altitudinal distribution, endemicity and conservation status.

Sp. ID	Species	Endemicity	Consv. St.	Altitude Range (m)				
				0-400	400-800	800-1200	1200-1800	>1800
1	Acacia melanoxylon	Exotic	NE					X
2	Acronychia pedunculata	Native	LC			X	X	X
3	Actinodaphne albifrons	Endemic	VU	X		X		X
4	Actinodaphne ambigua	Endemic	LC			X	X	X
5	Actinodaphne glauca	Endemic	NE				X	
6	Actinodaphne molochina	Endemic	EN					X
7	Actinodaphne speciosa	Endemic	EN				X	X
8	Adinandra lasiopetala	Endemic	EN				X	X
9	Aglaia apiocarpa	Native	LC			X		
10	Agrostistachys borneensis	Native	LC			X		
11	Agrostistachys hookeri	Endemic	LC	X				
12	Allophylus zeylanicus	Endemic	LC				X	
13	Alstonia macrophylla	Exotic	NE	X				
14	Ampelocissus indica	Native	NT		X			
15	Anisophyllea cinnamomoides	Endemic	NT	X	X	X		
16	Antidesma bunius	Native	LC				X	
17	Antidesma pyrifolium	Endemic	LC				X	
18	Aphanamixis polystachya	Native	VU			X		
19	Aporosa lanceolata	Endemic	LC	X	X			
20	Artabotrys zeylanicus	Native	LC	X				
21	Artocarpus nobilis	Endemic	LC	X	X			
22	Axinandra zeylanica	Endemic	VU	X	X			
23	Bhesa ceylanica	Endemic	LC	X	X			
24	Bhesa nitidissima	Endemic	LC	X		X		
25	Bridelia moonii	Native	LC	X				
26	Byrsophyllum ellipticum	Endemic	VU	X		X		
27	Calophyllum acidus	Native	NT			X		
28	Calophyllum bracteatum	Endemic	NT	X	X			
29	Calophyllum cf.trapezifolium	N/A	N/A			X		
30	Calophyllum cordato-oblongum	Endemic	EN	X	X			
31	Calophyllum moonii	Endemic	VU	X				
32	Calophyllum walkeri	Endemic	VU				X	X
33	Campnosperma zeylanica	Endemic	LC	X				
34	Canarium zeylanicum	Endemic	VU	X				
35	Canthium coromandelicum	Native	LC			X		
36	Carallia brachiata	Native	NT			X		
37	Carallia calytina	Endemic	EN			X		
38	Caryota urens	Native	LC		X			

Sp. ID	Species	Endemicity	Consv. St.	Altitude Range (m)				
				0-400	400-800	800-1200	1200-1800	>1800
39	Casearia esculenta	Native	LC			X		
40	Casearia thwaitesii	Native	VU					X
41	Celtis timorensis	Native	LC				X	
42	Cestrum aurantiacum	Exotic	NE				X	
43	Chaetocarpus castanocarpus	Native	LC	X	X	X		
44	Chaetocarpus coriaceus	Endemic	LC	X		X		
45	Chaetocarpus pubescens	Endemic	VU	X	X	X		
46	Chionanthus albidiflorus	Native	VU			X		
47	Cinnamomum cappara-coronde	Endemic	VU	X		X		
48	Cinnamomum dubium	Endemic	VU		X	X		
49	Cinnamomum litseifolium	Native	EN			X		
50	Cinnamomum ovalifolium	Endemic	VU				X	X
51	Cleistanthus ferrugineus	Endemic	LC		X			
52	Connarus championii	Endemic	NT	X				
53	Cryptocarya wightiana	Native	NT	X		X	X	
54	Cullenia rosayroana	Endemic	LC	X	X	X		
55	Cyathea hookeri	N/A	N/A					X
56	Cyathocalyx zeylanicus	Native	LC	X				
57	Dalbergia pseudo-sissoo	Native	LC	X	X			
58	Dichilanthe zeylanica	Endemic	VU	X				
59	Dillenia retusa	Native	LC	X				
60	Dillenia triquetra	Native	LC	X		X		
61	Dimocarpus longan	Native	LC	X				
62	Diospyros acuminata	Endemic	VU	X	X			
63	Diospyros albiflora	Native	EN	X				
64	Diospyros hirsuta	Endemic	VU	X	X			
65	Diospyros insignis	Native	LC	X		X		
66	Diospyros quaesita	Endemic	EN		X			
67	Diospyros Sp_KDN	N/A	N/A	X	X			
68	Diospyros srilankana	Endemic	VU	X				
69	Diospyros thwaitesii	Endemic	VU	X				
70	Diospyros toposia	Native	VU	X	X			
71	Diplospora erythrospora	Endemic	VU	X		X		
72	Dipterocarpus glandulosus	Endemic	EN	X				
73	Dipterocarpus hispidus	Endemic	VU	X	X			
74	Dipterocarpus zeylanicus	Endemic	NT	X				
75	Diyaminauclea zeylanica	Endemic	EN		X			
76	Donella lanceolata	Native	NT			X		
77	Drypetes sp	N/A	N/A		X			
78	Durio ceylanicus	Endemic	LC	X	X	X		

Sp. ID	Species	Endemicity	Consv. St.	Altitude Range (m)				
				0-400	400-800	800-1200	1200-1800	>1800
79	Dysoxylum championii	Endemic	VU	X	X	X		
80	Elaeocarpus amoenus	Endemic	VU	X				X
81	Elaeocarpus coriaceus	Endemic	EN				X	X
82	Elaeocarpus glandulifer	Endemic	VU	X	X	X		
83	Elaeocarpus montanus	Endemic	EN				X	X
84	Elaeocarpus subvillosus	Endemic	NT	X		X		
85	Enicosanthum acuminatum	Endemic	LC	X				
86	Erythrospermum zeylanicum	Endemic	LC	X		X		
87	Eugenia fulva	Endemic	CR(PE)			X		
88	Eugenia mabaeoides	Native	LC				X	
89	Eugenia mooniana	Native	LC		X			
90	Eugenia sripadaense	Endemic	CR				X	
91	Euphobiacea sp	N/A	N/A			X		
92	Eurya acuminata	Native	NT			X		
93	Eurya chinensis	Native	EN					X
94	Eurya nitida	Native	EN				X	
95	Fabacea sp	N/A	N/A		X			
96	Fagraea ceilanica	Native	NT			X		
97	Ficus drupacea	Native	LC		X			
98	Ficus microcarpa	Native	LC				X	
99	Ficus Sp	N/A	N/A			X		
100	Gaertnera rosea	Endemic	LC			X		
101	Gaertnera walkeri	Endemic	NT			X		
102	Garcinia echinocarpa	Native	VU			X	X	
103	Garcinia hermonii	Endemic	VU	X	X			
104	Garcinia morella	Native	NT	X	X	X		
105	Garcinia quaesita	Endemic	LC		X	X		
106	Garcinia spicata	Native	NT		X			
107	Garcinia stipulata	Endemic	EN			X		
108	Garcinia zeylanica	Endemic	EN	X	X			
109	Gironniera parvifolia	Native	LC	X		X		
110	Gomphandra coriacea	Native	VU				X	
111	Gomphandra tetrandra	Native	NT	X				
112	Gomphia serrata	Native	LC			X		
113	Goniothalamus gardneri	Endemic	VU			X		
114	Goniothalamus thwaitesii	Native	NT			X		
115	Gordonia elliptica	Endemic	EN				X	X
116	Gordonia speciosa	Endemic	EN				X	
117	Gyrinops walla	Native	VU	X				
118	Harpullia arborea	Native	VU	X				
119	Hedyotis dendroides	Endemic	NT					X

Sp. ID	Species	Endemicity	Consv. St.	Altitude Range (m)				
				0-400	400-800	800-1200	1200-1800	>1800
120	Homalium ceylanicum	Native	LC			X		
121	Hopea jucunda	Endemic	VU	X	X			
122	Hopea modesta	Endemic	EN	X				
123	Horsfieldia iryaghedhi	Endemic	VU		X			
124	Humboldtia laurifolia	Native	LC		X			
125	Hydnocarpus octandra	Endemic	LC	X		X		
126	Ilex walkeri	Native	LC					X
127	Isonandra zeylanica	Endemic	VU			X	X	
128	Ixora calycina	Native	VU				X	
129	Kokoona zeylanica	Endemic	EN		X	X		
130	Lijndenia capitellata	Endemic	VU		X			
131	Litsea glaberrima	Endemic	NT					X
132	Litsea ovalifolia	Endemic	NT				X	X
133	Macaranga indica	Native	LC		X			
134	Madhuca fulva	Endemic	VU	X	X			
135	Madhuca microphylla	Endemic	EN	X				
136	Madhuca moonii	Endemic	EN	X	X			
137	Maesa indica	Native	LC				X	
138	Magnolia nilagirica	Native	VU				X	X
139	Mallotus fuscescens	Endemic	LC	X				
140	Mangifera zeylanica	Endemic	LC	X	X		X	
141	Margaritaria cyanosperma	Endemic	VU	X				
142	Mastixia arborea	Native	VU				X	
143	Mastixia newsp	N/A	N/A			X		
144	Mastixia tetrandra	Native	LC	X	X			
145	Melicope lunu-ankenda	Native	LC			X	X	X
146	Meliosma pinnata	Native	VU				X	
147	Meliosma simplicifolia	Native	VU					X
148	Memecylon cf.fuscescens	N/A	N/A			X		
149	Memecylon giganteum	Endemic	EN	X				
150	Memecylon grande	Native	EN	X				
151	Memecylon Newsp	N/A	N/A			X		
152	Memecylon parvifolium	Endemic	VU				X	
153	Memecylon revolutum	Endemic	EN				X	
154	Memecylon rostratum	Endemic	NT	X	X			
155	Memecylon royenii	Endemic	LC	X				
156	Mesua ferrea	Native	LC	X	X			
157	Mesua thwaitesii	Endemic	LC		X			
158	Microtropis zeylanica	Endemic	NT				X	X
159	Myristica ceylanica	Native	VU	X	X	X		
160	Myristica dactyloides	Native	LC	X		X		

Sp. ID	Species	Endemicity	Consv. St.	Altitude Range (m)				
				0-400	400-800	800-1200	1200-1800	>1800
161	Myrsine thwaitesii	Native	NT					X
162	Myrsine wightiana	Native	VU			X		
163	Nargedia macrocarpa	Endemic	LC	X				
164	Neolitsea cassia	Native	LC	X		X		
165	Neolitsea fuscata	Endemic	VU				X	X
166	Nothapodytes nimmoniana	Native	NT				X	
167	Olea polygama	Native	LC				X	
168	Oncosperma fasciculatum	Endemic	VU			X		
169	Palaquium grande	Endemic	VU	X				
170	Palaquium hinmolpedda	Endemic	VU	X				
171	Palaquium laevifolium	Endemic	EN	X	X			
172	Palaquium pauciflorum	Endemic	EN	X				
173	Palaquium petiolare	Endemic	VU	X	X			
174	Palaquium rubiginosum	Endemic	VU				X	
175	Palaquium thwaitesii	Endemic	VU	X	X			
176	Palaquium zeylanicum	Endemic	CR	X				
177	Paracroton pendulus	Native	LC			X		
178	Paracroton zeylanicus	Endemic	LC	X				
179	Phoenicanthus coriacea	Endemic	EN				X	
180	Photinia integrifolia	Native	LC					X
181	Phyllanthus candolleanus	Native	LC				X	X
182	Phyllanthus nemoralis	Endemic	LC			X		
183	Pittosporum tetraspermum	Native	VU				X	X
184	Podadenia sapida	Endemic	VU		X			
185	Pometia pinnata	Native	NE		X			
186	Prunus ceylanica	Native	NT				X	
187	Psychotria dubia	Endemic	NT	X				
188	Psychotria nigra	Native	LC					X
189	Psydrax dicoccos	Native	LC	X			X	
190	Psydrax montana	Endemic	NT				X	
191	Putranjiva zeylanica	Endemic	LC		X			
192	Quassia indica	Native	VU	X				
193	Rhamnus arnottiana	Endemic	VU					X
194	Rhododendron arboreum	Endemic	VU					X
195	Rhodomyrtus tomentosa	Native	NT					X
196	Rourea minor	Native	LC		X			
197	Saprosma foetens	Native	LC				X	
198	Sarcococca brevifolia	Native	VU					X
199	Schefflera heterobotrya	Endemic	NT				X	
200	Schumacheria alnifolia	Endemic	EN				X	
201	Schumacheria castanifolia	Endemic	LC	X				

Sp. ID	Species	Endemicity	Consv. St.	Altitude Range (m)				
				0-400	400-800	800-1200	1200-1800	>1800
202	Scolopia crassipes	Endemic	LC				X	
203	Scolopia pusilla	Endemic	LC	X				
204	Scutinanthe brunnea	Native	EN			X		
205	Semecarpus coriacea	Endemic	VU				X	X
206	Semecarpus gardneri	Endemic	LC	X	X	X		
207	Semecarpus moonii	Endemic	VU	X				
208	Semecarpus newsp	N/A	N/A			X		
209	Semecarpus nigroviridis	Endemic	LC				X	
210	Semecarpus obovatus	Endemic	EN	X				X
211	Semecarpus parvifolia	Endemic	LC	X		X		
212	Semecarpus pubescens	Endemic	VU		X			
213	Semecarpus sp	N/A	N/A		X			
214	Semecarpus subpeltata	Endemic	VU	X	X			
215	Semecarpus walkeri	Endemic	LC	X	X	X		
216	Shorea affinis	Endemic	VU	X	X			
217	Shorea congestiflora	Endemic	VU	X	X			
218	Shorea cordifolia	Endemic	VU	X				
219	Shorea disticha	Endemic	VU	X				
220	Shorea dyeri	Endemic	VU	X				
221	Shorea gardneri	Endemic	VU			X		
222	Shorea hulanidda	Endemic	EN	X				
223	Shorea lissophylla	Endemic	VU	X	X			
224	Shorea megistophylla	Endemic	VU	X	X			
225	Shorea oblongifolia	Endemic	VU	X	X			
226	Shorea stipularis	Endemic	VU	X	X			
227	Shorea trapezifolia	Endemic	VU		X	X		
228	Shorea worthingtonii	Endemic	VU	X				
229	Shorea zeylanica	Endemic	EN			X		
230	stemonoporus sp	N/A	N/A			X		
231	Stemonoporus wightii	Endemic	EN	X				
232	Stemonurus apicalis	Endemic	NT	X	X			
233	Stemonurus sp	N/A	N/A	X				
234	Strobilanthes sexennis	Native	LC				X	
235	Strombosia ceylanica	Native	VU	X		X		
236	Swietenia macrophylla	Exotic	NE	X				
237	Symplocos bractealis	Endemic	EN				X	X
238	Symplocos cf. cochinchinensis	N/A	N/A				X	
239	Symplocos cochinchinensis	Native	LC				X	X
240	Symplocos coronata	Endemic	EN			X		
241	Symplocos elegans	Endemic	VU				X	X

Sp. ID	Species	Endemicity	Consv. St.	Altitude Range (m)				
				0-400	400-800	800-1200	1200-1800	>1800
242	Symplocos obtusa	Native	EN				X	X
243	Symplocos pulchra	Endemic	EN			X		
244	Syzygium alubo	Endemic	NT	X				
245	Syzygium amphoraecarpus	Endemic	NT	X				
246	Syzygium assimile	Native	LC				X	
247	Syzygium batadamba	Endemic	VU				X	X
248	Syzygium cordifolium	Endemic	VU	X	X			
249	Syzygium cylindricum	Endemic	LC		X	X		
250	Syzygium enasalwatta-new	N/A	N/A			X		
251	Syzygium firmum	Native	LC	X	X			
252	Syzygium kanneliyensis	Endemic	CR	X				
253	Syzygium micranthum	Endemic	LC			X		
254	Syzygium morningside	N/A	N/A				X	
255	Syzygium neesianum	Native	LC	X	X			
256	Syzygium New_PTD	N/A	N/A		X		X	
257	Syzygium revolutum	Endemic	LC				X	X
258	Syzygium rotundifolium	Endemic	LC					X
259	Syzygium rubicundum	Native	NT		X			
260	Syzygium wightianum	Endemic	LC		X			
261	Syzygium zeylanicum	Native	LC				X	
262	Tarenna flava	Native	LC				X	
263	Terminalia zeylanica	Native	LC		X			
264	Ternstroemia gymnanthera	Native	EN				X	
265	Timonius flavescens	Native	LC	X		X	X	
266	Turpinia malabarica	Native	LC				X	
267	Uncaria elliptica	Native	LC		X			
268	Urophyllum ceylanicum	Endemic	LC				X	
269	Urophyllum ellipticum	Endemic	LC			X		
270	Uvaria sphenocarpa	Endemic	LC		X			
271	Vaccinium leschenaultii	Native	VU					X
272	Vateria copallifera	Endemic	VU		X			
273	Ventilago gamblei	Native	LC		X			
274	Viburnum cylindricum	Native	EN					X
275	Wendlandia bicuspidata	Native	LC				X	X
276	Xylopia championii	Endemic	LC	X	X			

X (yellow)	Species present in one altitudinal class
X (cyan)	Species present in two altitudinal classes
X (red)	Species present in three altitudinal classes

CR(PE) – Critically-endangered, possibly extinct
CR – Critically-endangered
EN – Endangered
VU – Vulnerable
NT – Near-threatened
LC – Least-concern
NE – Not-evaluated
N/A – Not available

Table S2. Distribution and endemism of tree species found in altitudinal classes within tropical rainforests of Sri Lanka along an altitudinal gradient.

	Altitudinal range in which a species is found			
	one altitudinal class	2 Altitudinal classes	>2 Altitudinal classes	Total
Species total	176 (63.8%)†	82 (29.7%)	18 (6.5%)	276
Endemic spp.	83 (53.9%)	60 (39.0%)	11 (7.1%)	154 (55.8%)‡
Native spp.	73 (73.0%)	20 (20.0%)	7 (7.0%)	100 (36.2%)
Exotic spp.	4 (100.0%)	0 (0.0%)	0 (0.0%)	4 (1.4%)
Unidentified	16 (88.88%)	2 (11.11%)	0 (0.0%)	18 (6.5%)

†% of total species in each category in terms of the number of altitudinal ranges present; ‡% of total species for each category in terms of endemism.

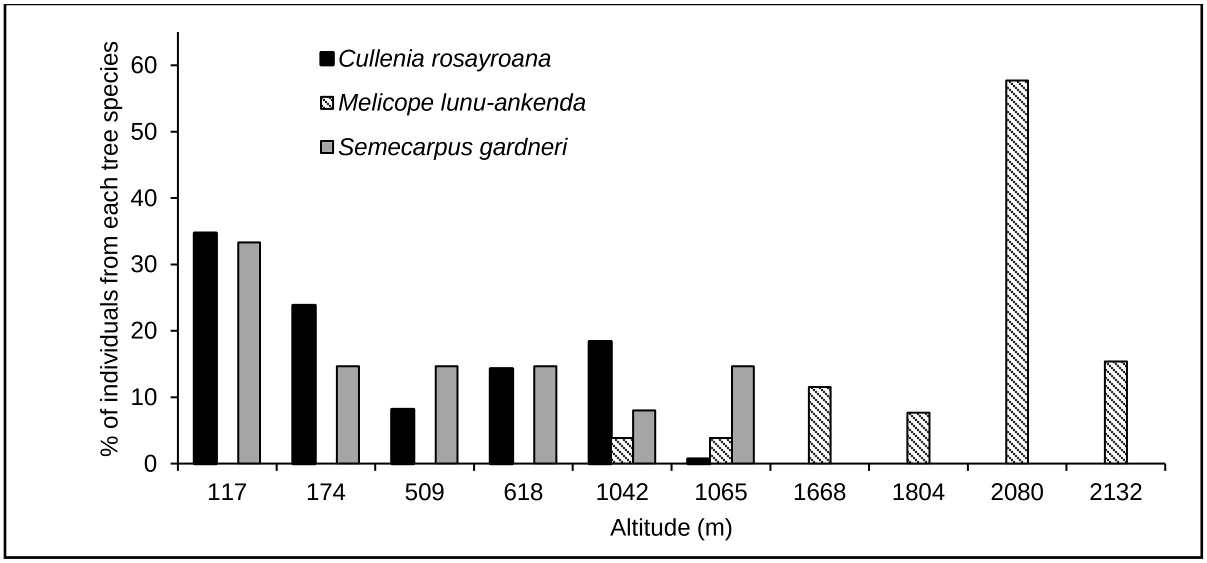

Figure S1. Distribution of individuals of the three tree species which were present in the greatest number of forest plots along the altitudinal gradient.

Table S3. Distribution and endemism of tree species found in altitudinal classes within tropical rainforests of Sri Lanka along an altitudinal gradient.

	Altitudinal range in which a species is found			
	one altitudinal class	2 Altitudinal classes	>2 Altitudinal classes	Total
Species total	176 (63.8%)†	82 (29.7%)	18 (6.5%)	276
Endemic spp.	83 (53.9%)	60 (39.0%)	11 (7.1%)	154 (55.8%)‡
Native spp.	73 (73.0%)	20 (20.0%)	7 (7.0%)	100 (36.2%)
Exotic spp.	4 (100.0%)	0 (0.0%)	0 (0.0%)	4 (1.4%)
Unidentified	16 (88.88%)	2 (11.11%)	0 (0.0%)	18 (6.5%)

†% of total species in each category in terms of the number of altitudinal ranges present; ‡% of total species for each category in terms of endemism.

Table S4 Number of individuals in different categories of endemism recorded in one-hectare permanent sampling plots within tropical rainforests of Sri Lanka along an altitudinal gradient.

No. of individuals	Endemic spp.	Native spp.	Exotic spp.	Unidentified	Total
1	15 (9.7%)[†]	21	-	6	42
2-10	63 (40.9%)	45	2	6	116
11-50	44 (28.6%)	17	1	5	67
51-100	17 (11.0%)	8	-	1	26
101-200	11 (7.1%)	8	1	-	20
> 200	4 (2.6%)	1	-	-	5
Total	154	100	4	18	276

[†]% of individuals out of the total of endemics.

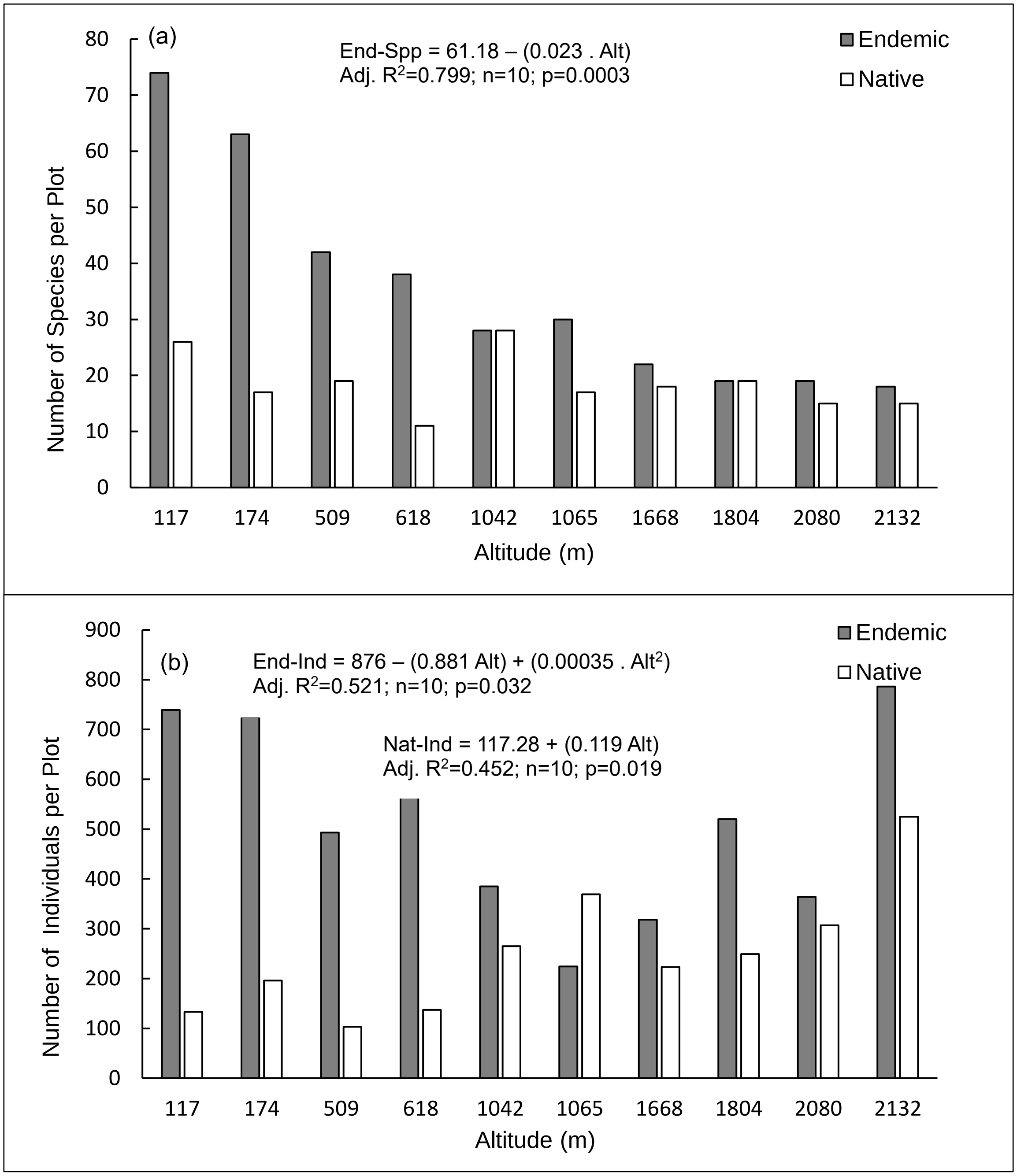

Figure S2. Altitudinal variation of the numbers of endemic and native tree species (a) and individuals (b) in forest plots along the altitudinal gradient. End-Spp – Number of endemic species; End-Ind – Number of endemic individuals; Nat-Ind = Number of native individuals; Alt – Altitude.

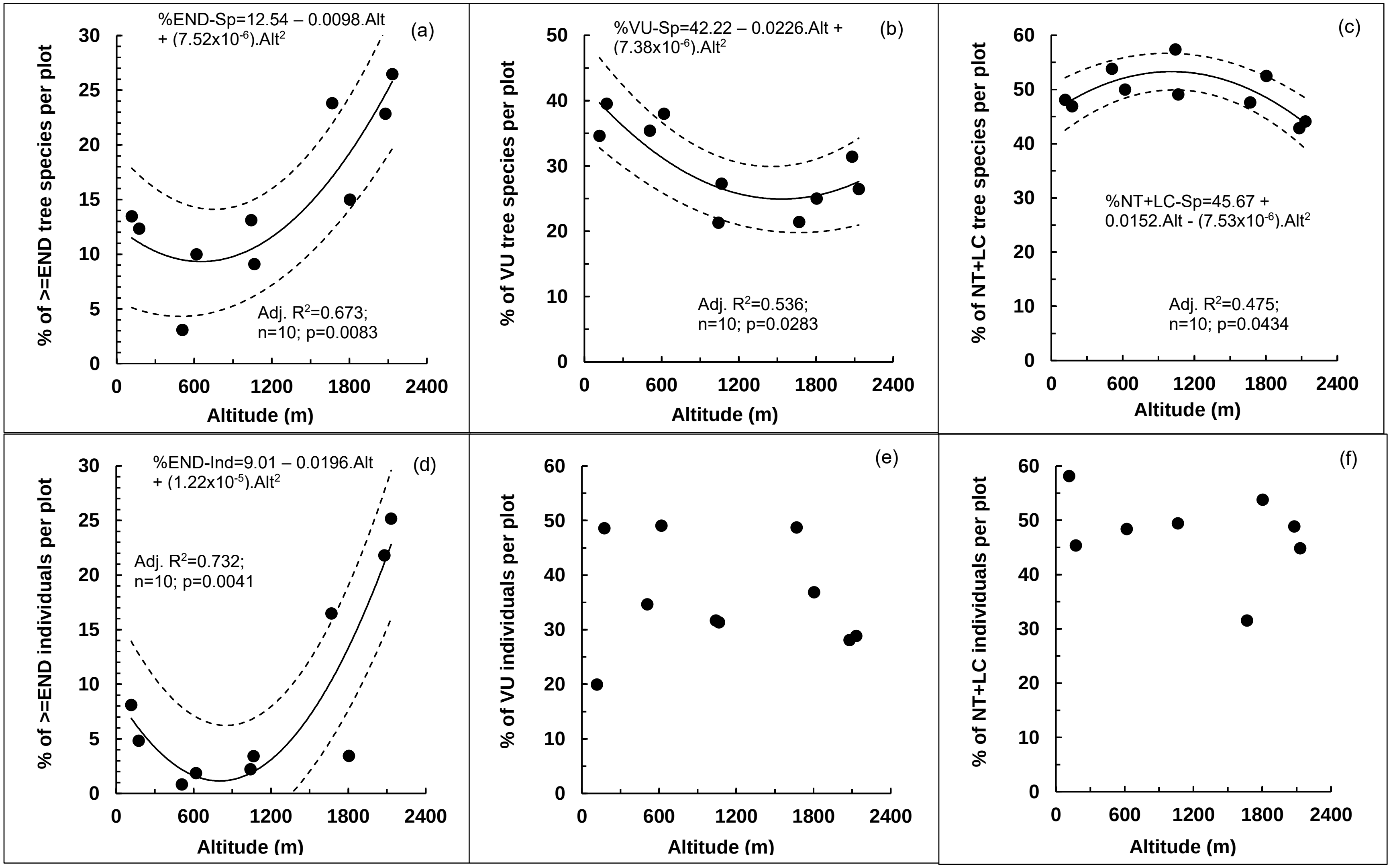

Figure S3. Variation with altitude of the per-plot percentages of: (a) endangered + critically-endangered + critically-endangered and nearly-extinct (≥END) species; (b) vulnerable (VU) species; (c) near-threatened + least-concern (NT+LC) species; (d) endangered + critically-endangered + critically-endangered and nearly-extinct (≥END) individuals; (e) vulnerable (VU) individuals; (f) near-threatened + least-concern (NT+LC) individuals in permanent sampling plots of selected tropical rainforests of Sri Lanka

Table S5. Partitioning of the scaled Chi-square in the canonical correspondence analysis of the influence of the matrix of selected climatic variables on the matrix of selected floristic variables of tropical rainforests along an altitudinal gradient.

	Inertia	Proportion
Total	0.08211	1.0000
Constrained	0.07090	0.8636
Unconstrained	0.01120	0.1364

Table S6. Accumulated constrained eigenvalues of canonical correspondence axes (CCA1 – CCA5) obtained from canonical correspondence analysis.

	CCA1	CCA2	CCA3	CCA4	CCA5
Eigen value	0.04932	0.0207	0.0008412	3.625e-05	1.124e-06
Proportion explained	0.69562	0.2920	0.0118635	5.112e-04	1.585e-05
Cumulative proportion	0.69562	0.9876	0.9994729	1.000	1.000

Table S7 Geographic and climatic details of the 1 ha permanent sampling plots in tropical rainforests of Sri Lanka along an altitudinal gradient

PSP†	Mean altitude (m asl)	Latitude (N)	Longitude (E)	Forest Type‡
KDN 1	117	6.24749	80.34071	Tropical Lowland rainforest
KDN 2	174	6.26090	80.35191	Tropical Lowland rainforest
PTD 2	509	6.39633	80.47070	Tropical Lowland rainforest
PTD 1	618	6.38141	80.47786	Tropical Lowland rainforest
ENS 1	1042	6.39433	80.59709	Tropical Lower montane forests
ENS 2	1065	6.39439	80.59565	Tropical Lower montane forests
RLG	1668	6.97619	80.58330	Tropical Montane Forest
HKG	1804	6.92725	80.81839	Tropical Montane Forest
PTG	2080	6.98197	80.77276	Tropical Montane Forest
HNP	2132	6.81459	80.80421	Tropical Montane Forest

†PSP – Permanent Sampling Plots; KDN 1, KDN 2 – Kanneliya Forest Reserve Plot 1 and 2; PTD-1, PTD-2 – Sinharaja-Pitadeniya Plot 1 and Plot 2; ENS-1, ENS-2 - Sinharaja-Enasalwatte Plot 1 and Plot 2; RLG - Rilagala Forest Reserve; HKG – Hakgala Strict Nature Reserve; PTG – Pidurutalagala Forest Reserve; HNP – Horton Plains National Park. T_{AV}, T_{MAX} and T_{MIN} – Long-term (1970-2000) annual average, maximum and minimum temperatures; R_F – Annual total precipitation.

Source: ‡Gunatilleke et al.[43].

Table S8. Classification of the disturbance status of the permanent sampling plots in tropical rainforests of Sri Lanka along an altitudinal gradient

Altitude (m)	PSP	Logging history	Removal of trees/litter traps during study period	Plot closer to forest boundary	Exotic tree species present	Proximity to human settlements	Suspected human activities (e.g. walking paths)	Overall disturbance status
117	KDN1	Yes	Few trees cut	Yes	Yes	Very close	Yes	High
174	KDN2	No	Few trees cut	No	No	No	Yes	Medium
509	PTD2	No	Litter traps removed	No	No	No	Yes	Medium
618	PTD1	No	No	No	No	No	Yes	Low
1042	ENS1	No	No	No	No	Medium	No	Low
1065	ENS2	No	No	No	No	Medium	No	Low
1668	RLG	Yes	No	Yes	No	Medium	Yes	High
1804	HKG	No	No	Yes	Yes	No	Yes	Medium
2080	PTG	No	No	Yes	Yes	No	Yes	Medium
2132	HNP	No	No	No	No	No	No	Low
